# Peroxisomes in brain development and function☆

**DOI:** 10.1016/j.bbamcr.2015.12.005

**Published:** 2015-12-11

**Authors:** Johannes Berger, Fabian Dorninger, Sonja Forss-Petter, Markus Kunze

**Affiliations:** Department of Pathobiology of the Nervous System, Center for Brain Research, Medical University of Vienna, Spitalgasse 4, 1090 Vienna, Austria

**Keywords:** Lipid metabolism, Plasmalogen, Zellweger spectrum disorder, D-bifunctional protein deficiency, X-linked adrenoleukodystrophy, Rhizomelic chondrodysplasia punctata

## Abstract

Peroxisomes contain numerous enzymatic activities that are important for mammalian physiology. Patients lacking either all peroxisomal functions or a single enzyme or transporter function typically develop severe neurological deficits, which originate from aberrant development of the brain, demyelination and loss of axonal integrity, neuroinflammation or other neurodegenerative processes. Whilst correlating peroxisomal properties with a compilation of pathologies observed in human patients and mouse models lacking all or individual peroxisomal functions, we discuss the importance of peroxisomal metabolites and tissue- and cell type-specific contributions to the observed brain pathologies. This enables us to deconstruct the local and systemic contribution of individual metabolic pathways to specific brain functions. We also review the recently discovered variability of pathological symptoms in cases with unexpectedly mild presentation of peroxisome biogenesis disorders. Finally, we explore the emerging evidence linking peroxisomes to more common neurological disorders such as Alzheimer’s disease, autism and amyotrophic lateral sclerosis. This article is part of a Special Issue entitled: Peroxisomes edited by Ralf Erdmann.

## 1 Introduction

Peroxisomes are single membrane-bound organelles, which harbor a variety of biochemical reactions and metabolic pathways that contribute to different physiological functions in eukaryotic organisms. Peroxisomes are found ubiquitously, but their number, shape and enzymatic content appear variable and differ between organisms and tissues and even upon changes in the environment [1]. In this review, we restrict the discussion to peroxisomal functions in the mammalian nervous system, with a specific focus on human physiology and pathophysiology supplemented by observations made in various mouse models. In mammals, peroxisomes contain around 50 different proteins [2], which exert a variety of catabolic and anabolic reactions as, for example, the degradation of very long-chain fatty acids (VLCFA)^1^, dicarboxylic acids, branched-chain fatty acids, or parts of the biosynthesis of ether phospholipids or specific polyunsaturated fatty acids [3].

**Fig.1:**
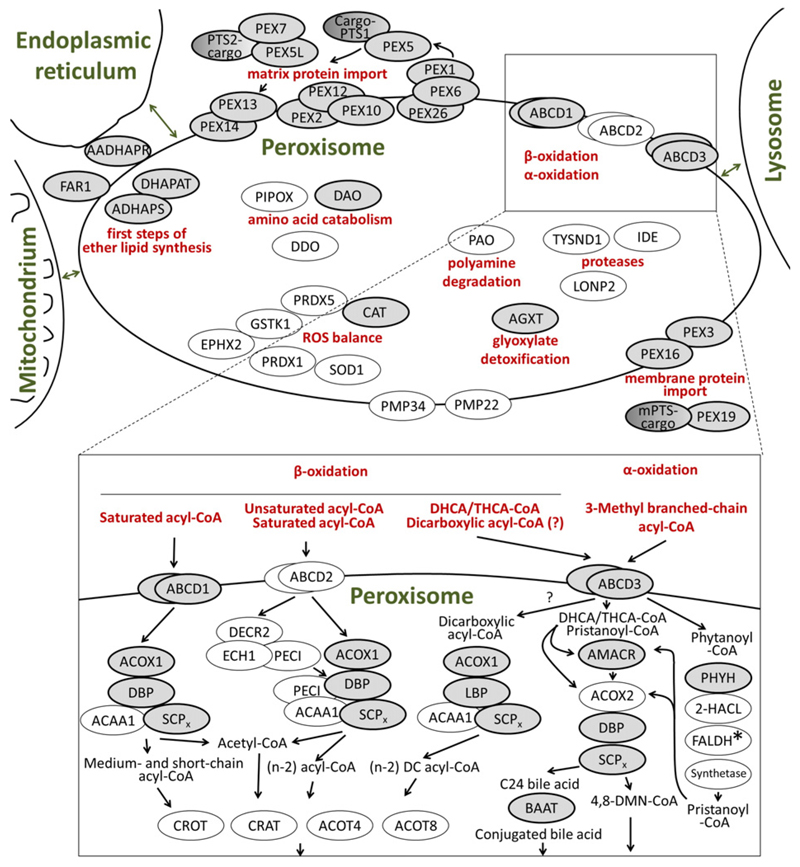
Schematic drawing linking peroxisomal disease-related proteins to individual metabolic pathways. *Upper part:* Proteins are grouped according to their function in biosynthetic or degradative metabolic pathways, ROS homeostasis, proteolytic activity, transport of metabolites across the peroxisomal membrane (ABCD and PMP proteins), and the import of matrix and membrane proteins (PEX proteins). *Ovals* represent proteins that are involved in peroxisomal functions (not complete); *gray ovals*, proteins for which mutations have been linked to a human disease (for full name see Table 1). The degradation of various fatty acids and bile acid precursors is symbolized by the frame depicting the homodimeric transporters (ABCD1–3) and the terms α- and β-oxidation, illustrated in more detail below. *Lower part:* Proteins are grouped into the degradation pathways for different activated fatty acids (fatty acyl-CoA: saturated, unsaturated, dicarboxylic, branched-chain) and the side chain shortening of di- and trihydroxycholestanoic acid (DHCA/THCA) during bile acid biosynthesis (all via β-oxidation) and the oxidative removal of one carbon unit from branched-chain fatty acids (α-oxidation). Several proteins are involved in the subsequent modification of the β-oxidation products, either by thiolytic cleavage (thioesterases, ACOT), substitution of CoA for carnitine (carnitine transferases, CRAT and CROT) or amidation of the CoA-activated side chain of bile acids (amino transferase, BAAT). FALDH*, two isoforms are known residing in peroxisomes and the ER, respectively, which precludes attribution of the linked disease, Sjögren–Larsson syndrome, to a particular variant. *Synthetase*, CoA-activation is essential for the link between α- and β-oxidation, but the exact enzyme has not yet been assigned. *PEX*, peroxin; *cargo-PTS1* and *PTS2-cargo*, representative peroxisomal matrix proteins harboring a PTS1 or PTS2 motif, respectively; *mPTS-cargo*, representative peroxisomal membrane protein harboring a motif for targeting of peroxisomal membrane proteins (mPTS). 4,8-DMN-CoA, 4,8-dimethylnonanoyl-CoA. Proteins not included in Table 1: *2-HACL*, 2-hydroxyacyl-CoA lyase; *ABCD2*, ATP-binding cassette transporter D2; *ACAA1*, acetyl-CoA acyltransferase 1; *ACOT4*, acyl-CoA thioesterase 4; *ACOT8*, acyl-CoA thioesterase 8; *ACOX2*, acyl-CoA oxidase 2; *CRAT*, carnitine O-acetyltransferase; *CROT*, carnitine O-octanoyltransferase; *DDO*, D-aspartate oxidase; *DECR2*, dienoyl-CoA reductase 2; *ECH1*, enoyl-CoA hydratase 1; *EPHX2*, epoxide hydroxylase 2; *GSTK1*, glutathione S-transferase kappa-1, *IDE*, insulin-degrading enzyme; *LONP2*, lon peptidase 2; *PAO*, polyamine oxidase; *PIPOX*, pipecolic acid oxidase; *PECI*, peroxisomal D3,D2-enoyl-CoA isomerase; *PMP22*, peroxisomal membrane protein of 22 kDa; *PMP34*, peroxisomal membrane protein of 34 kDa; *PRDX1*, peroxiredoxin 1; *PRDX5*, peroxiredoxin 5; *SOD1*, superoxide dismutase 1; *TYSND1*, trypsin domain-containing 1

The importance of peroxisomes for mammalian physiology is highlighted by the existence of a variety of severe inherited human diseases caused by the complete or partial loss of peroxisomal functions. These diseases have been subdivided into *peroxisome biogenesis disorders* (PBD), in which the formation of functional peroxisomes is disturbed, and *single enzyme and transporter deficiencies* lacking individual enzymatic activities that are performed by peroxisomes. Patients suffering from PBD show a broad spectrum of symptoms summarized as Zellweger spectrum disorders and rhizomelic chondrodysplasia punctata (RCDP) type 1. The genetic basis for each PBD is a mutation in one of 14 *PEX* genes, which encode proteins termed peroxins (PEX proteins or peroxisome biogenesis factors), which are involved in the biogenesis of the organelle (Table 1). All peroxisomal enzymes and membrane proteins contain a targeting signal, which is necessary and sufficient to mediate the interaction of the encoding protein with a receptor protein that translocates its cargo to peroxisomes and initiates the import. These processes are carried out by the PEX proteins (Fig. 1), which are either involved in the import of matrix proteins (PEX1, 2, 5, 6, 7, 10, 12, 13, 14, 26) or of membrane proteins (PEX3, 16 and 19) [4]. Soluble proteins harbor such peroxisome targeting signal (PTS) sequences either at their extreme C-terminus (type 1, PTS1) or close to their N-terminus (type 2, PTS2), whereas membrane proteins contain targeting signals for membrane proteins (mPTS). PTS1 is required for the interaction with the cytoplasmic receptor PEX5, PTS2 for the interaction with PEX7 and the mPTS for the interaction with PEX19. This is the reason why in Zellweger spectrum patients, on the cellular level, peroxisomes are either absent or empty (ghosts).

The symptoms of patients with peroxisomal single enzyme and transporter deficiencies have a broad heterogeneity, related to differences in the physiological role of the affected metabolic pathway or reaction [5]. In this group of inherited diseases, mutations have been identified in 13 different genes encoding peroxisomal enzymes and in two genes encoding peroxisomal transporter proteins (Table 1; Fig. 1).

The brain is the most elaborate organ of the mammalian body and consists of a variety of tissue-specific cell types: neurons (with hundreds of different subtypes), oligodendrocytes, astrocytes and microglia. These differ in structure and function but cooperate tightly to perform all the tasks attributed to the brain. Moreover, the structural complexity of brain organization requires a precisely coordinated developmental process to accomplish its proper formation. The central nervous system (CNS; brain and spinal cord) and the peripheral nervous system (PNS) use the same mechanisms for communication between neurons, which transmit information by chemical synapses between cells. In addition, efficient propagation of the electrical signal (action potential) along the nerve fibers is facilitated by myelin ensheathment of the axons. The complexity of the nervous system and the tight interaction of the involved cell types render this system susceptible to disturbances. Accordingly, metabolic dysfunction associated with a complete loss of all peroxisomal functions or of individual enzymatic reactions is often linked to perturbation of brain formation, function or maintenance. Thus, pathological aberrations of the nervous system are prominent features in most peroxisomal disorders; the most severe form of PBD has traditionally been designated “cerebro-hepato-renal syndrome” highlighting the apparent brain dysfunction in these patients. The brain pathology in peroxisomal disorders can be grouped into three major classes: i) abnormalities in neuronal migration or differentiation, ii) defects in the formation or maintenance of central white matter, and iii) post-developmental neuronal degeneration [6].

This review summarizes the current knowledge on the contribution of the various peroxisomal pathways to proper brain function with particular consideration of the different cell types of the nervous system.

## 2 Metabolic functions of peroxisomes

Peroxisomes harbor a variety of enzymes, which either serve to catalyze a single chemical reaction or cooperate with other peroxisomal enzymes in a series of coupled reactions constituting a complete metabolic pathway. A selection of these enzymes, which exert important peroxisomal functions in the context of the brain, is schematically depicted in Fig. 1. For further details on these metabolic pathways, the reader is referred to excellent previous reviews [3] [7].

A prominent example of such a metabolic pathway is the peroxisomal degradation of diverse fatty acids by β-oxidation (Fig. 1, lower part). Here, many different substrates are handled, such as straight-chain saturated VLCFA, unsaturated fatty acids, dicarboxylic acids and a subset of branched-chain fatty acids, but also the side chain of intermediates in bile acid biosynthesis (di- and trihydroxycholestanoic acid; DHCA and THCA) [7]. The β-oxidation cycle is a four-step reaction, executed by three enzymes: an acyl-CoA oxidase (ACOX1 or ACOX2), a bifunctional protein (DBP or LBP) and a thiolase (ACAA1 or SCPx), in which the paralogous/homologous enzymes show different extents of substrate specificity. Each cycle results in a shortening of the acyl-CoA backbone and the release of acetyl-CoA or propionyl-CoA (in case of branched-chain fatty acids). Auxiliary enzymes help to circumvent special properties of unsaturated or branched-chain fatty acids that would be incompatible with continuous β-oxidation. The substrates of β-oxidation are imported into peroxisomes in an activated form, as CoA-ester, via ATP-binding cassette (ABC) transporter proteins (ABCD1, ABCD2 and ABCD3) and the products are further processed either into carnitine esters by carnitine ac(et)yl-transferases (CRAT and CROT) or into free acids by thioesterases (ACOT4 and ACOT8) (Fig. 1, lower part). A subtype of branched-chain acyl-CoA (especially phytanic acid) first has to be oxidatively decarboxylated via the α-oxidation pathway [7]. This process involves hydroxylation of the carbon next to the carboxylate ester (by PHYH) and a subsequent oxidative cleavage to split off the carboxyl group by 2-hydroxyacyl-CoA lyase (2-HACL) releasing an acyl-aldehyde. The subsequent steps involve an oxidation of the aldehyde (fatty aldehyde dehydrogenase; FALDH) and an activation of the generated fatty acid by a still unknown acyl-CoA synthetase.

Furthermore, the early steps of ether phospholipid biosynthesis are exerted by peroxisomal enzymes (Fig. 1, upper part), which reside either inside (DHAPAT, ADHAPS) peroxisomes or at the outer side (FAR1, AADHAPR) [3]. This metabolic pathway consists of a series of reactions; the first, carried out by dihydroxyacetone phosphate acyltransferase (DHAPAT) combines dihydroxyacetone phosphate (DHAP) with a fatty acid, which is then exchanged for an long-chain alcohol by alkyl-DHAP synthase (ADHAPS). This long-chain alcohol is generated from another fatty acid by a fatty acyl-CoA reductase (FAR1) at the outer side of peroxisomes. Finally, the carbonyl group of the original dihydroxyacetone phosphate is reduced by alkyl/acyl-dihydroxyacetone phosphate reductase (AADHAPR) to enable further processing at the endoplasmic reticulum (ER).

Other peroxisomal enzymes can exert their function more independently (Fig. 1, upper part) such as the enzymes of the reactive oxygen species (ROS) detoxification system (peroxiredoxin 1/5, PRDX1/5; superoxide dismutase 1, SOD1; epoxide hydrolase, EPXH2; glutathione-S-transferase kappa 1, GSTK1 and catalase, CAT), which together prevent the accumulation of reactive compounds, as reviewed in [8]. Also several enzymes acting on amino acids and their derivatives (pipecolic acid oxidase, PIPOX; D-aspartate oxidase, DDO; D-amino acid oxidase, DAO; alanine:glyoxylate aminotransferase, AGXT) or other oxidative enzymes like polyamine oxidase (PAO) act in isolation [3]. Furthermore, several proteins with a protease domain have been found in peroxisomes (lon peptidase 2, LONP2; insulin-degrading enzyme, IDE; trypsin domain-containing 1, TYSND1) and some membrane proteins (peroxisomal membrane protein of 22 kDa, PMP22; and peroxisomal membrane protein of 34 kDa, PMP34), which transport a variety of smaller organic compounds such as nicotinamide-adenine-dinucleotides (NAD), CoA, or ATP [9].

The enzymes known to be dysfunctional in patients suffering from inherited peroxisomal disorders are distributed across these pathways (Fig. 1, gray ovals). However, the relative physiological contribution of each enzyme may differ drastically. Consequently, the pathological consequences of their functional loss range from very severe diseases, like D-bifunctional protein (DBP) deficiency (see chapter 5.2.2.), to diseases that affect selective tissues but not the brain, like AGXT deficiency causing primary hyperoxaluria type 1, which involves the kidneys [10].

## 3 Brain peroxisomes and how they differ from peroxisomes in other tissues

Although peroxisomes are present in all mammalian cell types, except for red blood cells, they contribute to the function of the CNS in specific ways. On the one hand, peroxisomes generate building blocks (intermediates) for the biosynthesis of complex lipids such as ether phospholipids, which are important components of myelin, the membrane processes of oligodendrocytes that ensheath and isolate axons. Moreover, peroxisomes exert the last step in the biosynthesis of the very long-chain polyunsaturated fatty acid docosahexaenoic acid (DHA; C22:6 n-3), which has important roles in the nervous system [11]. This fatty acid is enriched in phospholipids including ether phospholipids and, either directly or after enzymatic conversion to a variety of bioactive derivatives, plays an important role in signaling [12]. On the other hand, peroxisomes degrade toxic compounds that can either interfere with proper brain formation or damage brain structures (e.g., phytanic acid). Furthermore, peroxisomal enzymes degrade D-amino acids such as D-aspartate and D-serine, which modulate synaptic signaling by altering the efficiency of synaptic transmission (Fig. 3A, left panel).

In the brain, peroxisomes appear as electron-dense single membrane-bound organelles that have been detected in all neural cell types, namely in neurons [13], oligodendrocytes [14,15] and astrocytes [13] and microglia and endothelial cells [16]. Brain peroxisomes in general, and neuronal peroxisomes in particular, are smaller than peroxisomes from other tissues and, thus, were termed microperoxisomes [17]. However, for the sake of simplicity, we use the term peroxisomes for all structures within this review. In cultured cells from rat brain, punctate peroxisomal immunoreactivity was found in mixed glial cells and established oligodendrocyte cultures [18], as well as in astrocytes and neurons [19].

The distribution of peroxisomes in the brain has been investigated by different techniques such as cytochemical detection of enzymatic activities restricted to peroxisomes including 3-aminotriazol-sensitive precipitation of diamino-benzidine for catalase, conversion of D-proline for detection of DAO or of D-aspartate for DDO [20]. Moreover, immunohistochemistry, immunofluorescence microscopy and electron microscopy have been used. However, it is important to keep in mind that many studies examined the presence and abundance of a single peroxisomal protein, thus possibly detecting only a subset of peroxisome-positive cells. Therefore, it is necessary to combine the different investigations to obtain an insight into the accurate distribution and abundance of all peroxisomes in the brain. Comparison of DAO and catalase activity revealed that in the locus coeruleus of the rat brain, peroxisomes that stained positive for catalase activity were found in various cell types, whereas DAO activity-positive peroxisomes were restricted to astrocytes [13]. Similar results were obtained in the cerebrum and in the PNS [13]. In the cerebellum, punctate catalase immunoreactivity (characteristic of a peroxisomal localization) was predominantly observed in Bergmann glia (astrocytes), whereas in Purkinje cells, catalase appeared evenly distributed. This finding was recapitulated in explanted cells from the cerebellum, in which catalase appeared cytosolic (not enriched in peroxisomes) in calbindin-positive Purkinje cells but punctate in astrocytes, whereas the peroxisomal membrane protein PEX14 was found punctate in all cell types [21]. Moreover, the abundance of brain peroxisomes differs between brain areas. Although single membrane-bound structures – detectable with different methods to stain peroxisomes – can be found in most regions, some brain areas were reported to contain only modest numbers of peroxisomes [22]. However, peroxisome abundance also changes during development. In the human brain, catalase-positive neurons emerged early in evolutionary old structures such as the basal ganglia, the thalamus and the cerebellum (about 27–28 weeks of gestation), whereas in the frontal cortex, they appeared later (around 35 weeks of gestation) [15]. Similar observations were obtained when investigating the distribution of ACOX1 or thiolase (ACAA1) immunoreactivity [23]. In the deep white matter, catalase-positive glia appeared at 31–32 weeks of gestation, their appearance shifting from the deep to the superficial white matter with increasing age [15]. Interestingly, during rat brain development, peroxisomal activity (as represented by catalase activity) remained constant in the cerebral cortex (a typical gray matter region), whereas in the white matter, the activity changed over time with a clear peak accompanying the phase of myelination (during postnatal days 17–31) [24]. A similar increase in catalase activity was found in extracts from murine cerebellum and brain stem [25], whereas a systematic comparison by western blot analysis and catalase activity measurements found the maximum level two days after birth and at later timepoints, 15 and 49 days postnatally, the levels of peroxisomal enzymes remained comparable [19].

This change in protein abundance is reflected at the mRNA level, where the expression of genes coding for enzymes involved in the same metabolic pathways showed similar temporal profiles. In the murine brain, the mRNA levels of the peroxisomal β-oxidation enzymes (ACOX1, DBP, ACAA1a), the ABC transporters ABCD2 and ABCD3, and the enzymes involved in ether phospholipid biosynthesis increased after birth, reached a maximum during the first weeks and then declined. In contrast, the mRNAs for the enzymes involved in α-oxidation were not detected during the first postnatal weeks; and the *ABCD1* mRNA was most highly expressed in the embryonic brain [26–28]. This change in enzyme expression was confirmed in a systematic biochemical investigation of the abundance of peroxisomal enzymes and their activity during mouse brain development. In this study, it was found that peroxisomal activities decreased during postnatal development (P2, P15, P49), irrespective of whether the activity was normalized to the whole brain or to different brain regions (cerebellum, hippocampus, cortex) [19]. This is in agreement with previous findings in rat brain demonstrating that during the first two postnatal weeks, peroxisomes are more abundant than at later time points [22]. However, this general trend contrasts with the reported amount of DAO activity in astrocytes of rat cerebellum, which was only observed in adult rats, whereas no staining was observed in young animals (P3, P13, P16) [13]. This might indicate a more specific contribution of DAO in the adult brain, which could be linked to its function in the modulation of neuronal synaptic transmission (see chapter 6.1.).

**Fig.2:**
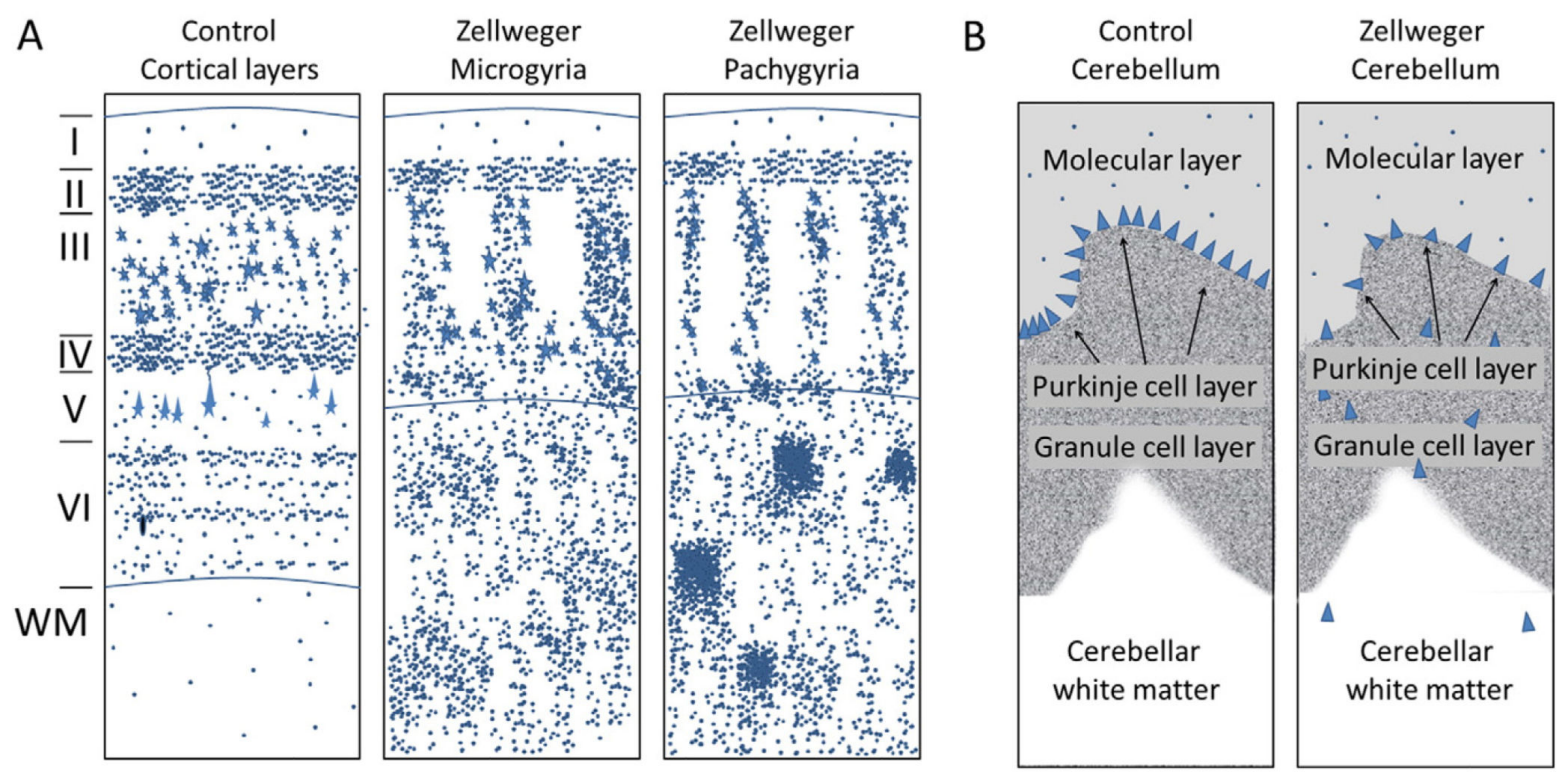
Schematic representation of neuronal migration defects in peroxisomal biogenesis disorders. (A) In the cerebral cortex (neocortex) of a healthy individual (left panel), the cell bodies of cortical neurons are localized in discrete layers. In comparison, the cortical lamination is severely disturbed and the border to the white matter in microgyric (middle panel) and pachygyric (right panel) brains of cases with Zellweger Syndrome is indicated (horizontal line). Similar abnormalities can also be found in cases of severe D-bifunctional protein deficiency. Roman numerals to the left correspond to normal cortical layers. *WM*, white matter. (B) In the cerebellum of a healthy individual (left panel), the Purkinje cells (blue triangles) are strictly arranged into a single cell-thick layer at the border of the molecular (outermost) layer and the thick granule cell layer. In Zellweger patients (right panel), many Purkinje cells are mislocalized to the granule cell layer and cerebellar white matter.

In the rat PNS, peroxisomes were described in Schwann cells, which represent the myelinating cells of the PNS, as well as in dorsal root ganglion satellite cells and, less abundantly, in neuronal somata [29]. In neurons of human dorsal root ganglia, peroxisomes were readily detected based on immunohistochemistry for ABCD1 [30]. During early stages of murine peripheral nerve (sciatic nerve) myelination, peroxisomes appear to be diffusely distributed in the myelin sheath of Schwann cells, whereas at later stages, peroxisomes were found to be enriched in the myelin loops of the paranodal region [14]. These axon-glia contact sites flank the nodes of Ranvier, substructures of myelinated neurons, in which highly abundant sodium channels in the axonal membrane enable depolarization and reinitiation of the action potential and thus permit the rapid saltatory propagation of the electrical signal across long distances (Fig. 2A, left panel) [14].

## 4 Peroxisomes, brain and oxidative stress

Oxidative stress is a cellular state characterized by a high level of ROS such as hydrogen peroxide (H_2_O_2_) or superoxide anions (O_2_·^–^), which are considered to be mediators of the toxic effects associated with oxidative stress. This state often arises as side effect of cellular disturbances and has been amply described in connection with general peroxisomal dysfunction, but also upon specific loss of an individual peroxisomal function [31]. An increase in the concentration of ROS can originate either from overproduction by one or more cellular producers (individual enzymes or whole organelles), a reduction of the detoxifying activity exerted by protective proteins (catalase, glutathione peroxidase, superoxide dismutase, peroxiredoxin) or a shortage of scavenging molecules that normally buffer the emerging ROS molecules (e.g., glutathione, vitamin C and E) [32].

Peroxisomes are known to house a variety of oxidases generating H_2_O_2_ and ROS, but they also enclose various ROS-detoxifying enzymes such as catalase, GSTK1, PRDX5 and SOD1 to limit the detrimental effects of local production [8]. However, this detoxification system can be overloaded. Artificial local production of ROS inside peroxisomes can induce apoptosis, which can be rescued by ectopic overexpression of peroxisomal detoxifying enzymes [33]. Exogenously added palmitate can stimulate H_2_O_2_ production in peroxisomes of insulin-producing cells [34] and exogenous application of VLCFA to a neuronal cell line induces oxidative stress and mitochondrial damage [35]. Prolonged hyperactivity of peroxisomes has also been linked to the overproduction of H_2_O_2_ in the liver of acyl-CoA oxidase-deficient mice [36]. Surprisingly, in patients suffering from acatalasemia, an inherited peroxisomal disorder caused by the loss of functional catalase, no neurological involvement or brain abnormalities have been described, although this enzyme plays such a prominent role in oxygen metabolism [37,38].

Moreover, peroxisomes are involved in the biosynthesis of plasmalogens, which have been suggested as scavenger molecules for H_2_O_2_ and ROS [39]. However, this effect is partially disputed, because plasmalogen-deficient mice do not show signs of increased oxidative stress [40]. This issue has been extensively covered in previous reviews [41,42]. Furthermore, the absence of one or more peroxisomal functions can indirectly increase the level of intracellular ROS, because under such conditions, the accumulation of particular compounds such as VLCFAs could be linked to disturbances in mitochondrial integrity, which secondarily increases the production rate of ROS [43,44].

## 5 Brain dysfunctions in inherited peroxisomal disorders

### 5.1 Brain pathology under conditions of generalized peroxisome deficiency in man and mice

This section focuses on the brain pathology in disorders of peroxisome biogenesis or assembly, collectively known as peroxisome biogenesis disorders (PBD). In these disorders, peroxisomes are not formed normally, typically leading to deficiency of the entire spectrum of peroxisomal functions. Thus, the observed pathology cannot be attributed to individual peroxisomal metabolic pathways but rather reflects the importance of the entire organelle for brain development and maintenance. In addition, genetically engineered mouse models with tissue- or cell type-specific inactivation of peroxisome biogenesis demonstrate the importance of peroxisomes for the different brain cell types, as well as the significance of peroxisomal functions in peripheral tissues for proper brain development.

#### 5.1.1 Brain pathology in human patients with PBD

The PBD are divided into two types, i) Zellweger spectrum disorders and ii) rhizomelic chondrodysplasia punctata (RCDP) type 1. In our current understanding, the clinical syndromes constituting the Zellweger spectrum (MIM #601539)^2^ are the Zellweger syndrome, neonatal adrenoleukodystrophy, and infantile Refsum disease, which describe a clinical spectrum of decreasing severity [45]. These were originally described as independent disorders, long before the biochemical and molecular bases of these disorders were understood [45]. In 1992, the first gene defect associated with a PBD was identified [46]. By now, mutations in 13 different *peroxin* (*PEX*) genes (*PEX1, PEX2, PEX3, PEX5, PEX6, PEX10, PEX11b, PEX12, PEX13, PEX14, PEX16, PEX19, PEX26*) have been described in patients of the Zellweger spectrum [45]. Patients with mutations in *PEX7* are grouped into PBD because more than one peroxisomal pathway is affected, although the peroxisomal structure remains intact. However, these patients have different clinical symptoms than Zellweger spectrum patients and are clinically not distinguishable from patients suffering from isolated disorders of ether phospholipid synthesis; hence, the associated brain pathology will be discussed in Section 5.5.

The identification of PBD complementation groups and their genetic basis has revealed that there are no clear boundaries between Zellweger syndrome, neonatal adrenoleukodystrophy and infantile Refsum disease, as they can all be caused by mutations in the same gene. However, a genotype-phenotype correlation has been described for *PEX* gene mutations [45]. The nature and location of the mutations determine whether the mutated peroxin can still contribute to the import machinery and allows residual metabolic functions of the peroxisomes in these patients. In recent years, increasing numbers of patients have been described with a later onset of the disease [47]. In accordance, also the neurological manifestations vary from primarily neurodevelopmental alterations in the most severe phenotypes to mainly degenerative abnormalities in the milder cases [48].

In patients with Zellweger syndrome, the most prominent feature of the brain pathology is a malformation of the cortex, which has been attributed to neuronal migration defects. The abnormalities in the cytoarchitecture of the cerebral cortex are usually bilateral and approximately symmetrical [49,50]. In these patients, often a local thickening of small convolutions (gyri) on the surface of the brain occurs around the central sulcus (centrosylvian pachygyria), causing a reduced depth of the fissions/involutions. Moreover, in these areas, an excess of local convolutions on the surface of the brain is observed (polymicrogyria). The cytoarchitectonic pattern of the cerebral cortex is disturbed in the microgyric and pachygyric areas (Fig. 2A). These abnormalities were characterized in terms of the relative positions of specific neuronal subsets and the patterns of neuronal arrangements into radial groups (Fig. 2A) [50]. In the polymicrogyric cortex, typically a fusion of the molecular layers is associated with a modified distribution of medium to large pyramidal cells originating from the deep cortex. This causes a decrease in the numbers of neurons in the outer layers (layer II and layer III) of the cortex; instead, these neurons are located in the deep cortex and within heterotopias of subcortical white matter (Fig. 2A) [6]. Less severe cerebral migratory abnormalities were reported in neonatal adrenoleukodystrophy [51]. To date, no migration defects have been described in infantile Refsum disease, the least severe form of the Zellweger spectrum. Another striking morphological aberration linked to neuronal migration defects is the heterotopic localization of Purkinje cells in the cerebellar white matter (Fig. 2B) [6,49,50].

In addition to these migration defects, within their first year of life, all patients with Zellweger syndrome display white matter abnormalities in the CNS, which have been observed by histological analyses and brain magnetic resonance tomography (MRT) studies [52–55]. Because myelination is still ongoing during this early period, it cannot be clearly established, whether the lack of peroxisomal functions causes abnormal myelination, early demyelination or both processes simultaneously [56]. Neuropathological examination of brains obtained from three cases of neonatal adrenoleukodystrophy revealed a severe degeneration of the white matter involving both hemispheres of cerebrum and cerebellum, while the axons were preserved [57]. In the cerebellum of one case, overabundance of reactive astrocytes in the white matter was associated with perivascular cuffs of mononuclear cells [57]. Heterotopic Purkinje cells were found to be aggregated in irregular clumps in the subcortical areas of the cerebellar cortex in two of the three cases [57]. In some cases, mild initial symptoms are later followed by severe CNS demyelination and death of the patient [53,55,56]. In the mildest forms of the Zellweger spectrum, patients can survive into adulthood [47,58,59]. In a study of 19 patients (16–35 years old) with such a mild Zellweger spectrum disorder, magnetic resonance imaging (MRI) revealed white matter abnormalities in nine of the patients. These abnormalities were restricted to the cerebellar hilus of the dentate nucleus and/or the peridentate region [47]. During infancy, four of these patients suffered from hypotonia, five from failure to thrive, 12 had a visual handicap due to retinal degeneration and eight presented with hearing impairment. During childhood, all 19 patients had a moderate to severe developmental delay as well as a reduction/loss of visual and hearing abilities and seven did not achieve structured speech. The predominant neurological symptom in the adult patients was a gait disorder, caused by different combinations of cerebellar syndrome, pyramidal tract dysfunction and peripheral neuropathy. Interestingly, at the time of diagnosis, 17 of these patients had a blood metabolite profile typical of a peroxisomal disorder; but at later time points the concentration of many originally accumulating metabolites had declined and, in some patients, even a complete normalization was observed. In particular, the levels of intermediates of bile acid biosynthesis (DHCA, THCA) and of pipecolic acid declined during the observed time period in many patients, whereas VLCFA and plasmalogen levels normalized only in some. This implies that, based on plasma metabolites linked to peroxisomal function, some of these patients would have escaped the diagnosis of a Zellweger spectrum disorder. Other studies reported normal VLCFA levels in plasma of late-onset patients with a *PEX2* mutation [59] or normal plasmalogen levels in plasma of patients with *PEX16* mutations [58]. Accordingly, for the Zellweger spectrum, separate MIM numbers have been assigned according to the severity of phenotypes (Table 1). These findings indicate that the level of peroxisome-related metabolites in plasma may not necessarily reflect the level of accumulation in tissues. This is of great relevance for the interpretation of alterations of plasmalogen levels in plasma of patients with more common neurological diseases (see Section 6.4.).

#### 5.1.2 Brain pathology in mouse models of the Zellweger spectrum disorders

Currently, several mouse models of the Zellweger spectrum disorders are available, which are represented by mice with targeted deletions in the genes encoding the peroxins PEX2, PEX5 or PEX13 [60–62]. Recently also a knock-in mouse model carrying a missense mutation in the *Pex1* gene (*Pex1*-G844D) was reported, which recapitulates the most frequent mutation in the human Zellweger spectrum disorders with a milder pathology [63]. As *Pex7*-deficient mice represent a model for RCDP type 1, but not for Zellweger spectrum disorders, we discuss this model in the context of ether phospholipid deficiency (Section 5.5.). Moreover, mouse models with *Pex11α* [64] and *Pex11β* [65] deficiencies have been generated.

The phenotype of mice with *Pex2, Pex5* and *Pex13* deficiency resembles the severe form of human Zellweger syndrome. These mice are born alive, but are growth-retarded and severely hypotonic. Moreover, they do not feed and die within 67#x2013;24 h after birth [60–62]. When the *Pex2* mutation was maintained on a mixed genetic background (Swiss Webster × 129Svev), about 25% of the *Pex2*^−/−^ pups survived for one to two weeks [66]. Furthermore, postnatal survival could be improved by oral bile acid application (9% alive after 30 days) [67]. In all mice with a global peroxisome deficiency (*Pex2, Pex5* and *Pex13* deficiency), a reduced thickness of the neocortical plate was observed, which reflects abnormal lamination that has been linked to impaired neuronal migration and increased cellular density in the underlying white matter [60–62]. Also cerebellar malformation was explored in all three models of PBD revealing abnormalities in cerebellar foliation. However, because the cerebellum develops largely postnatally in mice, a detailed characterization of cerebellar development was only possible in the longer surviving (Swiss Webster × 129Svev) *Pex2*^−/−^ mice [68,69]. These studies revealed multiple anomalies affecting the interaction of climbing fibers, granule cells and Purkinje cells and, thus, the cerebellar circuitry. The number of granule cells was reduced due to defects in their migration from the external to the internal granule cell layer and increased apoptotic cell death. The Purkinje cells displayed stunted dendrite trees with abnormal branches and spine morphology. The disturbed dendritic spine compartmentalization reflected a delayed arborization and translocation of the climbing fibers from the inferior olivary nucleus (the major excitatory input to the Purkinje cells from the caudal medulla). In addition, progressive axonal swellings along Purkinje cell axons indicated ongoing dystrophic, neurodegenerative processes.

With regard to the *Pex11*-related mouse models, it should be noted that in contrast to the other peroxins, the PEX11 family members act as membrane elongation factors during peroxisome proliferation [70]. Whereas PEX11α appears not to be essential for the formation of functional peroxisomes, the absence of PEX11β leads to several pathological features shared by the mouse models of Zellweger syndrome, including neuronal migration defects, enhanced neuronal apoptosis, developmental delay, hypotonia and neonatal lethality [65]. As the import of peroxisomal proteins is not impaired in this mouse model, no accumulation of VLCFA and only a slight decrease in plasmalogen levels were detected in the brain [65]. The mechanism, by which *Pex11β* deficiency causes Zellweger-like symptoms, in spite of the mild metabolic defects, remains to be resolved.

#### 5.1.3 The importance of peroxisomal Junctions for individual cell types of the brain

The power of mouse genetics provides an opportunity to discriminate the contribution of peroxisomal functions from different cell types to brain development and function. The conditional inactivation of selected genes in specific cell types or tissues has been used to generate mice with a deficiency in all peroxisomal functions restricted to subsets of brain cells. By crossing mice with a “floxed” *Pex5* gene (*Pex5* flanked by *loxP* recombination sites), which are susceptible to the removal of the DNA region between the *loxP* sites by the cyclization recombinase (Cre), and mice expressing Cre in a subset of cells, mouse lines have been generated, in which peroxisomes are selectively absent from different compartments of the CNS according to the specificity of the *Cre*-driving promoters. When using mice, which express Cre under the *nestin* promoter (*Nestin-Cre* driver mice), inactivation of *Pex5* occurs in all neural precursor cells at embryonic stages, but not in the microglia lineage. This results in the ablation of peroxisomal functions in the vast majority of neurons, astrocytes and oligodendrocytes of mice already at prenatal stages [71]. These *Nestin-Pex5*^−/−^ mice appear normal at birth, but develop substantial growth retardation after the first postnatal week. Progressive motor impairments ensue, resulting in lethargy and death before six months of age. In these mice, peroxisome-dependent metabolite levels were deranged (increased VLCFA, decreased plasmalogen levels) in the brain at late embryonic stages, but were normal in the liver. In the developing brain, a defect in neuronal layer formation in the cerebral cortex was observed indicative of neuronal migration defects and, postnatally, delayed cerebellar development including immature foliation and dendritic arborization of Purkinje cells [72]. Marked hypomyelination was detected already during the second to third postnatal week (probably due to insufficient formation of myelin) and was found in all brain regions (later probably also due to demyelination), together with axonal loss, reactive astrocytes as well as activated microglia and macrophages [73]. Also brain-specific (*Nestin-Cre*-dependent) inactivation of *Pex13* in mice resulted in a similar phenotype with impaired cerebellar development, neuronal cell death, astrogliosis and microgliosis as well as signs of mitochondria-mediated oxidative stress [74]. Similarly, selective knockout (KO) of *Pex5* in oligodendrocytes by using *Cnp-Cre* drivers had severe consequences for the adult brain [75]. Interestingly, no developmental defects were observed at birth or after two months although CNPase is expressed in progenitors before myelination as well as in adult oligodendrocytes. However, young adult mice gradually developed impaired motor function and premature death due to axonal degeneration, progressive subcortical demyelination and neuroinflammation, starting at two to six months of age. In contrast, peroxisome ablation in projection neurons of neocortex and hippocampus, obtained with *Nex-Cre* driver mice [76], or in astrocytes obtained with *GFAP-Cre* drivers [76], had no obvious deleterious effect on brain development or function. This was surprising, because *Pex5* deletion in astrocytes resulted in accumulation of VLCFA as well as reduced plasmalogen levels in the brain. Taken together, these studies indicate that in the murine brain, peroxisomes are most crucial for oligodendrocytes and the myelin compartment.

However, also the selective loss of peroxisomal functions in hepatocytes of the liver, obtained by *α-fetoprotein-Cre* driver mice [72], results in brain abnormalities including defects in cerebral neuronal migration and cerebellar development (hypotrophy, increased apoptosis, immature foliation, delayed granule cell migration and stunted Purkinje cells). This finding is further corroborated by observations in *Pex5*-deficient mice (ubiquitous KO), in which liver-specific ectopic expression of *Pex5* [77] resulted in partial rescue of the brain defects. These studies indicate a role of brain-extrinsic effects (effects originating from outside the brain) in CNS development in peroxisomal disorders. The mechanisms are not resolved but Faust and collaborators showed that bile acid treatment can partially restore the cerebellar anomalies in *Pex2*-deficient mice [69]. This treatment partially compensates for the lack of mature C24 bile acids in this mouse model and, thus, restores intestinal absorption of dietary lipids. However, it is unclear, whether the beneficial effect of this treatment on postnatal CNS/cerebellum development is due to an improved metabolic state of the pups because of increased lipid absorption, or prevention of steatorrhoea and cholestasis, or whether the addition of mature bile acids reduces the synthesis of bile acid precursors, which might impair CNS development. However, the absence of developmental problems in the CNS of racemase-deficient mice, in which bile acid precursors accumulate as well [78], renders an exclusive effect of bile acid precursors quite unlikely.

### 5.2 Brain pathology in peroxisomal β-oxidation disorders in humans and mice

As peroxisomes fulfill a variety of metabolic functions, which are concomitantly ablated upon inactivation of peroxisome biogenesis (in human patients suffering from Zellweger syndrome or in *Pex*-deficient mice), the attribution of particular aspects of brain pathology cannot be traced back to a single pathway such as β-oxidation. The investigation of single enzyme and transporter deficiencies and mouse models lacking individual peroxisomal enzymes or transporter proteins allows a comparison of the physiological consequences of a selective loss of individual metabolic pathways for brain function. However, even these conditions have limitations, because metabolic pathways such as the peroxisomal β-oxidation handle many different substrates, which renders a direct correlation between the loss of an enzymatic activity and a class of substrates impossible. Peroxisomal β-oxidation can degrade VLCFA, branched-chain fatty acids, bile acid intermediates, long-chain dicarboxylic acids and polyunsaturated fatty acids like tetracosahexaenoic acid (C24:6), which undergoes one cycle of β-oxidation in peroxisomes to produce DHA (C22:6). Moreover, fatty acid-like compounds with signaling activity such as prostaglandins and leukotrienes and some classes of xenobiotics are degraded in peroxisomes [3]. Notably, some activities in the β-oxidation pathway can be executed by more than one isoenzyme (Fig. 1). Human peroxisomes harbor two acyl-CoA oxidases, two bifunctional enzymes and two thiolases, whereas murine peroxisomes are equipped with three acyl-CoA oxidases, two bifunctional enzymes and three thiolases. As most of the human isoenzymes of the peroxisomal β-oxidation have different substrate specificities, some enzyme deficiencies lead to a rather selective accumulation of specific peroxisomal β-oxidation substrates, as will be discussed in the corresponding sections below.

#### 5.2.1 Peroxisomal acyl-CoA oxidase deficiency

Patients with an inactivating mutation in *ACOX1* lack peroxisomal acyl-CoA oxidase activity, which is responsible for the degradation of saturated VLCFA, polyunsaturated fatty acids and dicarboxylic acids, but not branched-chain fatty acids or bile acid intermediates (Fig. 1). Still, in many respects, the clinical presentation of acyl-CoA oxidase deficiency (formerly pseudoneonatal adrenoleukodystrophy) resembles Zellweger spectrum disorders, notably neonatal adrenoleukodystrophy [79]. Most patients show neonatal onset of hypotonia, seizures, failure to thrive, hepatomegaly, psychomotor retardation, sensory deafness, absent reflexes, and visual loss with retinopathy and extinguished electroretinograms [80]. Patients may show some early delay in motor development with a typical regression by 273x2013;3 years of age. Brain imaging (MRT and/or CT) revealed cerebral and/or cerebellar white matter abnormalities in all investigated patients in a study involving 12 subjects, of whom three showed neocortical dysplasia [79]. As for the Zellweger spectrum disorders, recently several cases of acyl-CoA oxidase deficiency with less severe clinical phenotypes were reported, which progressively developed neurological symptoms in later childhood [81].

To recapitulate the human disease, a mouse model with generalized *Acox1* deficiency was generated [82], in which VLCFA accumulate. In *Acox1*^−/−^ mice, marked peroxisome proliferation in the liver was observed and accompanied by an increase in H_2_O_2_ concentration, leading to the development of hepatic adenomas and carcinomas at 15 months of age [36]. However, no brain pathology has been described for these mice. To date, neither patients with mutations in *ACOX2* nor *Acox2* or *Acox3*-deficient mice have been described.

#### 5.2.2 D-Bifunctional protein deficiency

In humans, two peroxisomal bifunctional proteins exist: D-bifunctional protein (DBP; alternatively termed multifunctional protein 2; encoded by *HSD17B4*) and L-bifunctional protein (LBP; alternatively termed multifunctional protein 1; encoded by *EHHADH*), both having a catalytic 2-enoyl-CoA hydratase activity and a (3R)-hydroxyacyl-CoA dehydrogenase activity (Fig. 1). All known human patients with bifunctional protein deficiency harbor mutations in the *HSDI7B4* gene [83], whereas no patients with a mutation in the *EHHADH* gene, encoding LBP, have yet been identified. The existence of two enzymatic domains allows the classification of mutations based on the location and the nature of the mutation. Mutations affecting both domains or destabilizing the protein are classified as DBP deficiency type I, those affecting only the hydratase domain as DBP deficiency type II, and those solely affecting the dehydrogenase unit as DBP deficiency type III. However, as both enzymatic steps are essential for peroxisomal β-oxidation, the complete loss of both activities as well as of the individual enzymatic activities causes neurodevelopmental abnormalities and death within the first two years of life [83]. The severe form of DBP deficiency mimics Zellweger syndrome in all aspects including cranio-facial dysmorphism, neuronal migration defects (similar to that depicted in Fig. 2A) and premature death [84]. Also, demyelination of the central white matter is present [83].

Similar to the Zellweger spectrum disorders, recently also patients with unexpected phenotypes of DBP deficiency were identified using next generation sequencing [85]. These patients presented with ovarian dysgenesis, hearing loss, and ataxia comparable to Perrault Syndrome (MIM #233400) demonstrating clinical overlap of DBP deficiency and the genetically heterogeneous Perrault Syndrome [85]. One of the documented patients, who was 27 years old at the last examination [85], had normal levels of VLCFA and phytanic acid [86]. Normal serum VLCFA levels have been reported also in other patients with later clinical onset of DBP deficiency. The correct diagnosis in these cases was initiated by neuroimaging or whole exome sequencing [87,88]. This further demonstrates that peroxisome-related neurological deficits and the level of metabolites linked to peroxisomal functions do not necessarily correlate when measured in the blood of patients.

A mouse model of DBP deficiency (here termed *Mfp2* deficiency) has been described, in which VLCFA accumulate specifically in brain, and the degradation of branched-chain fatty acids as well as the maturation of bile acid precursors were disturbed [89]. *Mfp2*-deficient mice appear quite normal at birth but are severely growth-retarded during the lactation period. Their life span is markedly reduced with a part of the population dying early (at around two weeks of age) [89] while the rest lives for up to six months [90]. Notably, this is much longer than the survival of *Pex5*-deficient mice (6–24 h) [60]. Moreover, *Mfp2*-deficient mice do not show signs of neurodevelopmental abnormalities such as migration defects at early time points [90] [91]; but later on, these mice develop cerebellar aberrations and axonal loss, which is reflected by motor impairment and lethargy [92]. Thus, in contrast to the human disorders, where Zellweger syndrome and DBP deficiency are clinically very similar, their respective mouse models are remarkably different. Interestingly, the brain pathology of *Mfp2*-deficient mice resembles the conditional *Nestin-Pex5* mouse model (see 5.1.3), in which functional peroxisomes are absent from all neural cell types of the CNS [71].

#### 5.2.3 2-Methylacyl-CoA racemase deficiency

The enzyme 2-methylacyl-CoA racemase (AMACR) inverts the steric configuration at the position next to the thioester, resulting in the conversion of (2R)-methyl branched-chain fatty acids into (2S)-methyl branched-chain fatty acids. Only branched-chain acyl-CoAs such as pristanic acid or bile acid intermediates with the 2-methyl branch in the S configuration are substrates for peroxisomal β-oxidation. Accordingly, pristanic acid and the bile acid intermediates DHCA and THCA, but not VLCFA, accumulate in *AMACR*-deficient patients (Fig. 1) [93]. The phenotype of patients with *AMACR* deficiency (MIM #614307) often involves adult-onset sensory neuropathy [94] and late-onset cerebellar ataxia [95]. Occasionally, other symptoms and types of pathology have been described such as white matter anomalies [96], relapsing encephalopathy [97] and a more complex adult phenotype including peripheral neuropathy, epilepsy, bilateral thalamic lesions, cataract, pigmentary retinopathy and tremor [98]. Finally, some patients had cholestatic liver disease in the first neonatal weeks [99].

The generation of a racemase-deficient mouse model has been described [78], but so far only the pathological features of peripheral lipid metabolism have been investigated [100]. However, upon phytol supplementation of the diet, the mice developed severe pathology in the brain after 40 days, including demyelination and activation of astroglial cells [101].

#### 5.2.4 SCPx deficiency and gene redundancy of peroxisomal thiolase activity and the consequences for brain function

In man, two enzymes with thiolase activity, acetyl-CoA acyltransferase 1 (ACAA1) and sterol carrier protein X (SCPx), are present in peroxisomes (Fig. 1). However, only for SCPx, the thiolase required for the breakdown of branched-chain fatty acids, a single patient with a deficiency of the enzyme (MIM #613724) has been described so far [102]. Among other clinical features, this adult patient presented with dystonic head tremor and spasmodic torticollis; and cranial MRI showed bilateral hyperintense signals in the thalamus, butterfly-like lesions in the pons and lesions in the occipital region [102].

In the mouse, three enzymes with thiolase activity exist: two closely related proteins (96% amino acid sequence identity) encoded by the differentially regulated genes *Acaa1a* and *Acaa1b*, and SCPx. In a classical gene KO model, *Acaa1b* deficiency showed a very mild phenotype and hardly any accumulation of VLCFA, indicating a compensatory effect from *Acaa1a* and/or *Scpx* in mice [103]. In *Scpx*-deficient mice, methyl-branched-chain fatty acid catabolism is impaired resulting in a mild phenotype under standard conditions [104]. However, high phytol diet treatment led to a much more severe phenotype, in which the mice rapidly lost body weight and acquired an unhealthy appearance and inactivity, reduced muscle tone, ataxia and trembling [104].

### 5.3 Brain pathology in X-linked adrenoleukodystrophy

Three peroxisomal ATP-binding cassette (ABC) transporters, ABCD1, ABCD2 and ABCD3, mediate the translocation of activated fatty acids and probably other compounds across the peroxisomal membrane, in order to get metabolized within the peroxisomes (Fig. 1). The abundance of these transporter proteins varies between cell types and tissues [28,105–107]. Inherited defects in the *ABCD1* (formerly *ALD*) gene are the genetic basis for X-linked adrenoleukodystrophy (X-ALD; MIM #300100) [108]. X-ALD is the most common peroxisomal disorder with an estimated combined male and female incidence between 1:16,800 [109] and 1:30,000, with similar incidence rates across the world [110]. Human ABCD1 transports CoA-activated saturated straight-chain VLCFA across the peroxisomal membrane for further degradation by the peroxisomal β-oxidation machinery (Fig. 1) [111]. Upon ectopic overexpression in yeast, ABCD1 can mediate the transport of a broader spectrum of substrates [112], and overlapping substrate specificities have been demonstrated for the three peroxisomal ABC transporters [113–115]. Because ABCD2 and ABCD3 as well as the peroxisomal β-oxidation enzymes are intact in X-ALD, only saturated straight-chain VLCFA accumulate, but to a variable extent in different cell types and tissues. This selective substrate transport deficiency as well as the overlapping functions of the peroxisomal ABCD transporters explains why, in contrast to DBP deficiency and acyl-CoA oxidase deficiency, some X-ALD patients can remain pre-symptomatic through more than five decades, even in the complete absence of ABCD1 transporter activity. In addition to the impaired degradation of VLCFA, probably also increased fatty acyl chain elongation of long- to very long-chain acyl-CoA esters contributes to the accumulation of VLCFA (in particular C26:0) in X-ALD. Leading studies of Stephan Kemp's group suggest an important role of the rate-limiting enzyme in this process, elongation of very long-chain fatty acids 1 (ELOVL1), in the homeostasis of VLCFA in X-ALD [116,117]. Among the seven known ELOVL family members, which have different chain length selectivity, ELOVL1 favors saturated and monounsaturated CoA-activated fatty acids with a chain length of 20 to 24 carbons [118,119]. Indeed, upon knockdown of *ELOVL1* mRNA in X-ALD fibroblasts, the storage of C26:0 decreased significantly [116].

Although X-ALD does not involve any developmental defect or delay, it is characterized by remarkable clinical heterogeneity. The main phenotypes are adrenomyeloneuropathy (AMN) and cerebral ALD (CALD), the devastating inflammatory and demyelinating form of X-ALD [120]. Both phenotypes can occur within the same kindred [121] and no general genotype–phenotype correlation exists for the severity in X-ALD [122–125]. Adrenal insufficiency represents another major pathological aspect in X-ALD, which often represents the initial symptom and affects 80% of male patients before adulthood but is rare in heterozygous female patients [126]. Virtually all male patients with mutations in the *ABCD1* gene eventually develop AMN, a slowly progressive myelopathy with typical onset in the third or fourth decade of life. The earliest symptoms are usually urge incontinence and sensory disturbances in the legs followed by spastic gait. The major neuropathological feature in AMN is a distal dying-back axonopathy, which involves the dorsal columns and corticospinal tracts in the lower thoracic and lumbar regions [127], as well as the more proximal segments of the corticospinal tracts in the internal capsule [128]. The peripheral nerves are also involved, with primary axonal degeneration in most AMN patients [129]. Evidence of myelopathy or peripheral neuropathy was recently observed in more than 80% of women carrying heterozygous *ABCD1* mutations and older than 60 years. Thus, female patients develop symptoms similar to those in male AMN patients but at later age [130,131].

**Fig.3:**
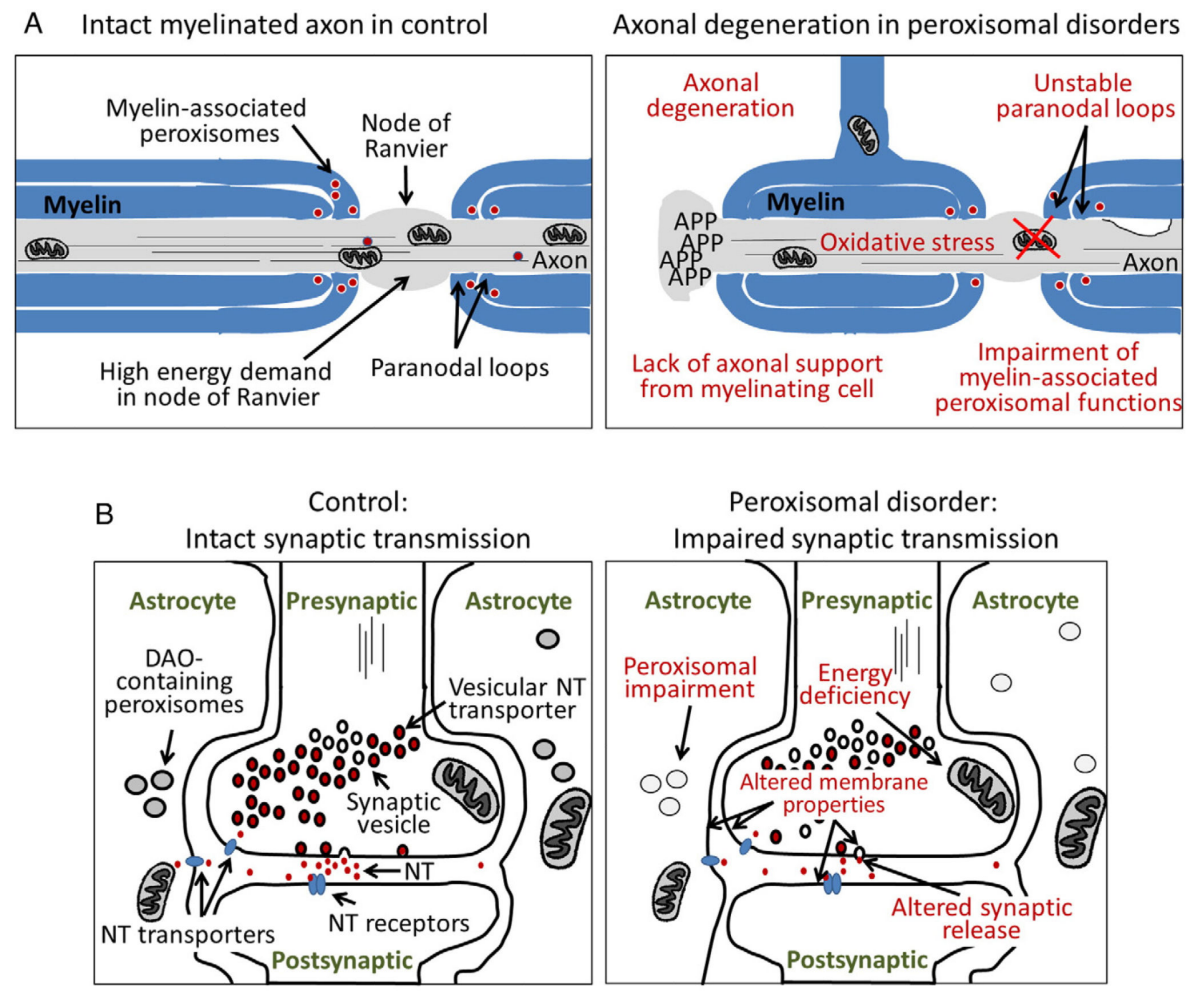
Schematic representation of abnormalities of myelinated axons and synaptic transmission in peroxisomal deficiencies. (A) The left panel shows a myelinated axon at the level of a node of Ranvier in a healthy control. The myelin sheath of oligodendrocytes (in the CNS) or Schwann cells (in the PNS) surrounds and isolates the axon, except at the node of Ranvier allowing depolarization of the neuronal membrane and propagation of electrical signals. Note that a multitude of ion channels and Na^+^/K^+^-ATPases (not indicated) are located at the node of Ranvier and entail a high energy demand. In the right panel, different pathological features are indicated that may contribute to the axonal degeneration frequently observed in peroxisomal disorders, for example, adrenomyeloneuropathy (the late-onset variant of X-ALD). A scenario can be envisaged, where peroxisomal dysfunction and abnormal accumulation of lipid metabolites in myelinating cells lead to unstable paranodal loops and a loss of axonal support resulting in energy deficits and oxidative damage in the axons and progressive axonal degeneration. (B) A normal synapse with the surrounding astrocytes is depicted (left panel), representative for a synapse of any neurotransmitter. D-Amino acid oxidase is indicated for its role in D-serine degradation at e.g. glutamatergic synapses. The right panel shows several possible disturbances of synaptic function (red text) that could lead to altered neurotransmission, as predominantly described in ether lipid deficiency. *NT*, neurotransmitter; *DAO*, D-amino acid oxidase

In the human brain, based on immunohistochemical detection, ABCD1 is predominantly expressed in oligodendrocytes, microglia, astrocytes and endothelial cells but not in most neurons, with the exception of a few regions: hypothalamus, basal nucleus of Meynert, periaqueductal gray matter and the locus coeruleus [16,30]. Furthermore, ABCD1 is also highly expressed in dorsal root ganglia, where the neuronal cell bodies of the afferent sensory axons are located, which degenerate in AMN [30]. Thus, pathophysiological involvement is suggested for neurons as well as for oligodendrocytes in the case of axonopathy. By electron microscopy, mitochondrial abnormalities have been observed in neurons of AMN patients [132]. The mitochondrial abnormalities have been confirmed and are believed to be a major pathogenic factor contributing to neurodegeneration in AMN (Fig 3A). Cytosolic deposits of crystalline lamellar lipids were observed in brain macrophages, Schwann cells of peripheral nerves, adrenocortical cells, and Leydig cells of the testes. Cholesterol esters of VLCFA constitute a major component of these crystalline structures. Furthermore, it has been reported that VLCFA can disturb calcium homeostasis and cause mitochondrial dysfunction in neuronal cell cultures as well as toxicity to oligodendrocytes [133].

About 60% of male X-ALD patients develop CALD, the fatal cerebral demyelinating form of the disease. This can occur either in childhood, most commonly between 5 and 10 years of age, before onset of AMN (about 35%) or later in adolescence or adulthood, often on the background of AMN (35%). In children, the first symptoms are emotional lability, hyperactive behavior, school difficulties, impaired auditory discrimination and difficulties in vision [134]. These early clinical symptoms are not specific and often the correct diagnosis of X-ALD is delayed. This phase is followed by a rapidly progressing neurological decline, typically leading to a vegetative state or death within two to five years. For a male patient born with an *ABCD1* mutation, it cannot be predicted whether or when the cerebral form will develop. It is currently hypothesized that the cerebral inflammatory phenotype results from a “second hit”, superimposed on the axonal pathology [120]. Based on the lack of a genotype–phenotype correlation in X-ALD, it is likely that a combination of genetic, epigenetic and environmental factors plays an essential role as trigger for the development of CALD. This is also supported by the development of different clinical phenotypes in monozygotic twins [135,136] and the observation that moderate head trauma can initiate cerebral demyelination in AMN patients [137,138]. In magnetic resonance images of the brain of CALD patients, a typical enhancement of the border of the demyelinating lesion is visible after gadolinium administration reflecting an increased permeability of the blood brain barrier due to a marked inflammatory reaction [139]. In this active region, infiltration of macrophages, CD4+ and CD8 + cytotoxic T cells, as well as activated microglia and astrocytes can be observed [140]. This severe neuroinflammation probably causes the loss of oligodendrocytes, which die by cytolysis rather than by apoptosis [140]. Expression of proinflammatory cytokines such as tumor necrosis factor α, interleukin (IL)-1, IL-2, IL-6, IL-12 and interferon-γ and chemokines is increased [141–143]. The importance of microglia in the disease mechanism is supported by the observation of a zone within the perilesional white matter, immediately beyond the actively demyelinating lesion edge, lacking microglia [144]. This might be due to the migration toward the active age of the lesion. In the same study, clusters of activated and apoptotic microglia were detected within the subcortical white matter [144]. Another characteristic of the inflammation in CALD is the resistance to anti-inflammatory therapy. Based on our recent observations, we have suggested that this is due to the intrinsic metabolic defect of macrophages and microglia in X-ALD; these cells cannot degrade VLCFA, which they have taken up by phagocytosing of myelin debris (particular rich in VLCFA in X-ALD), and then fail to support normal immunological brain function [107, 123]. Based on this intrinsic defect, the continuous metabolic stress in the macrophage/microglia populations could also be the reason why only in rare cases a spontaneous arrest of brain inflammation occurs. Allogenic hematopoietic stem cell transplantation [145,146] and autologous stem cell-based gene therapy [147] can arrest the inflammatory demyelinating process with a typical delay of 12–18 months, which has been attributed to the slow replacement of microglia with bone marrow-derived phagocytes [147,148]. It must be noted that, due to the rapid disease progression, hematopoietic stem cell transplantation is only beneficial when performed at an early stage of disease.

Transcriptomic analyses of X-ALD brain tissue have indicated that already in AMN patients, a proinflammatory status prevails [149]. Musolino and coworkers recently demonstrated that inactivation of *ABCD1* induces significant alterations in the brain endothelium via c-MYC and may thereby contribute to the increased trafficking of leukocytes across the blood–brain barrier [150]. As the cell-autonomous (intrinsic) metabolic defect in the monocyte–macrophage lineages is also present in AMN patients, together with blood–brain barrier abnormalities, it appears reasonable that this fragile system is predisposed for converting to the inflammatory form of X-ALD, triggered by a broad spectrum of genetic and environmental factors.

In 1997, three independent groups had generated mouse models for X-ALD by targeted inactivation of the *Abcd1* gene [151–153]. Although the biochemical phenotype of X-ALD (i.e., accumulation of saturated VLCFA) was well replicated in all three models, *Abcd1*-deficient mice did not experience brain inflammation and demyelination as seen in humans with CALD. However, after 18 months of age, these mice start to develop a late-onset, mild motor behavior phenotype with resemblance to AMN including sciatic nerve conduction abnormalities and mild signs of axonopathy and myelin instability in the spinal cord [154]. Interestingly, *Abcd1* deficiency could further enhance microglia activation and axonal degeneration in mice with mild myelin abnormalities caused by the loss of the myelin-associated glycoprotein [155]. It is intriguing that also *Abcd1/Abcd2* double-deficient mice do not develop brain inflammation or demyelination [156], in spite of the finding that *Abcd1/Abcd2* double-deficient peritoneal macrophages are metabolically much more severely affected than those from single transporter-deficient mice [157]. Also in these mice, the neuropathology is restricted mainly to axonopathy in the spinal cord and, with the major contribution from *Abcd2* deficiency, the dorsal root ganglia resulting in a sensory neuropathy [156]. However, in the double mutant mice these abnormalities develop about six months earlier than upon sole *Abcd1* deficiency.

In addition to exploring the effects of therapeutics aimed at normalizing VLCFA levels *in vivo*, these mouse models have been applied to further characterize the mitochondrial damage noticed in X-ALD. The mitochondrial disturbances are probably not simply secondary effects due to VLCFA accumulation itself [158] but more complex, involving oxidative stress and cell type- and tissue-specific mechanisms that are of particular importance for axonal degeneration in the spinal cord [43,159]. Interestingly, lipoxidative damage was observed early (at three months of age) in the spinal cord of *Abcd1*-deficient mice, long before the onset of any neuropathological or motoric abnormalities were detected [160].

Evidence for the role of oxidative stress in plasma of X-ALD patients comes from an increased level of thiobarbituric acid reactive species (TBA-RS) reflecting induction of lipid peroxidation, as well as a decrease of plasma total antioxidant reactivity, indicating a deficient capacity to rapidly handle an increase of ROS [161]. Additional evidence comes from the finding of decreased levels of total and reduced glutathione, which were associated with high levels of oxidized glutathione, in lymphocytes of X-ALD (predominantly AMN) patients [162]. Also, decreased plasma thiols and a high level of carbonyls were found, additionally supporting the idea of oxidative stress – at least in blood cells – in X-ALD patients [162]. Encouraging results were obtained from a study applying an antioxidant cocktail consisting of vitamin E, N-acetylcystein and lipoic acid to aging *Abcd1*-deficient mice; this dietary treatment was sufficient to prevent the onset of locomotor disability and axonal damage [163]. In line with these findings, also oral administration of pioglitazone, an agonist of peroxisome proliferator-activated receptor γ (PPARγ) and inducer of mitochondrial biogenesis and respiration, was able to prevent mitochondrial damage and oxidative stress in *Abcd1*-deficient mice and could rescue the locomotor disability and axonal damage in the *Abcd1/Abcd2* double-deficient mouse model [164].

It has previously been suggested that mitochondrial dysfunction and oxidative stress within the axons are, at least partially, secondary to dysfunctions in the oligodendroglia/myelin compartment resulting in compromised support of axonal integrity [14,165,166]. In this context, it is noteworthy that mice with a sole defect in a myelin protein, such as proteolipid protein or 2′,3′-cyclic nucleotide phosphodiesterase, display axonal dysfunction without demyelination in the spinal cord and brain [167,168]. Most interestingly, mice with oligodendroglia-selective peroxisome deficiency (see also Section 5.1.3.) can also be considered as a phenocopy model for the inflammatory form of X-ALD, recapitulating widespread axonal degeneration, progressive subcortical demyelination and a proinflammatory milieu with B and T cell infiltration of brain lesions [75].

Our current hypothesis envisions the inability to degrade VLCFA combined with the increased elongation of VLCFA, in particular in oligodendrocytes and neurons, as the primary cause of the late-onset, slowly progressing, chronic myeloneuropathy in AMN (Fig. 3A). In heterozygous X-ALD females, a similar disorder develops, but with a later onset and slower progression; most likely random X-inactivation of the intact *ABCD1* copy leads to chimerism with a variable extent of *ABCD1*-deficient cells.

### 5.4 Brain pathology in α-oxidation deficiency in man and mice

With respect to the peroxisomal fatty acid catabolism, 2-methyl branched-chain fatty acids can directly enter the peroxisomal β-oxidation pathway, whereas 3-methyl branched-chain fatty acids cannot. Instead, 3-methyl branched-chain fatty acids can either be degraded by ω-oxidation (for review see [169]) or by peroxisomal α-oxidation (see Fig. 1). Among the enzymes involved in the α-oxidation pathway, only phytanoyl-CoA hydroxylase (PHYH) has been associated with a human disorder. Mutations in the *PHYH* gene have been established as the genetic cause for classical Refsum disease (MIM #266500) [170, 171]. The 3-methyl branched-chain fatty acid phytanic acid, solely taken up from dietary sources, accumulates in patients with Refsum disease. Because the disease is caused by the cumulative load of phytanic acid in tissues, the age of onset varies from early childhood to the fourth decade of life [56]. Refsum disease is characterized by progressive retinitis pigmentosa culminating in blindness, peripheral neuropathy and cerebellar ataxia [172]. When phytanic acid levels in the plasma remain low due to dietary restriction or repeated plasmapheresis the progression of the symptoms can be arrested [173,174]. Because phytanoyl-CoA hydroxylase is imported into peroxisomes via its PTS2 motif in a PEX7/PEX5L dependent manner, the α-oxidation pathway is also impaired in RCDP type 1 (*PEX7* deficiency, see Section 5.5) and in RCDP type 5 (deficiency in PEX5L).

A mouse model for Refsum disease has been generated by targeted disruption of the *Phyh* gene [175]. Because standard mouse chow is very low in branched-chain fatty acids, *Phyh*-deficient mice have an unremarkable phenotype. However, dietary supplementation with 0.25% phytol (the precursor of phytanic acid) for three weeks or 0.1% phytol for six weeks caused ataxia, reflecting Purkinje cell loss and astrogliosis in the cerebellum, and peripheral neuropathy, as revealed by nerve conduction velocity measurements [175].

### 5.5 Nervous system pathology in ether phospholipid deficiency in man and mice

So far, the biological functions of ether phospholipids (also simply termed ether lipids), especially in the CNS, have not been fully unraveled. However, many clues have been derived from the pathology of ether phospholipid-deficient mice and men. In humans, the lack of these lipids causes the lethal disease RCDP, an autosomal recessively inherited disorder with an estimated incidence of about 1:100,000. On a genetic basis, several different types are distinguished; RCDP type 1 (MIM #215100) is evoked by mutations in the gene encoding PEX7 [176–178], the cytosolic receptor for peroxisomal import of PTS2-containing proteins, whereas RCDP type 2 (MIM #222765) and type 3 (MIM #600121) are caused by mutations in the genes of the first two enzymes for biosynthesis of ether phospholipids, dihydroxyacetone phosphate acyltransferase (DHAPAT, DAPAT; encoded by the *GNPAT* gene) and alkyl-dihydroxyacetone phosphate synthase (ADHAPS; encoded by the *AGPS* gene), respectively [179,180]. Due to the fact that not only ether lipid biosynthesis but also peroxisomal α-oxidation is impaired in RCDP type 1, it is classified as a PBD rather than a pure ether lipid biosynthesis defect (see also Section 5.1). However, as the different RCDP types are clinically indistinguishable and the clinical manifestations of Refsum disease (see Section 5.4) are considerably less severe than that of RCDP type 1, we will cover RCDP type 1 in the present section, which focuses exclusively on ether phospholipids. The contribution of α-oxidation deficiency to the clinical phenotype may, however, be more prominent in RCDP type 1 patients with a milder disease course (see below) [181,182]. In addition, it can be speculated that some *PEX7* mutations affect proteins with certain PTS2 variants more strongly than others, thereby shifting the impact of the affected pathways on pathology. Recently, two additional subtypes of RCDP were identified based on the strong reduction of plasmalogen levels in the patients and the similarity of their symptoms with “classical” RCDP. First, the disorder of three patients with a deficiency in *FAR1*, the gene coding for fatty acyl-CoA reductase 1, which generates the fatty alcohols necessary to form the ether bond of the 1-alkyl chain in ether phospholipid biosynthesis [183], was categorized as RCDP type 4 [80]. Second, the disease in patients with mutations specifically affecting the long isoform of PEX5, PEX5L, which is required for efficient transport of cargo-loaded PEX7 to peroxisomes [184], was designated RCDP type 5 [185].

The pathology in all subtypes of RCDP has been more or less exclusively assigned to the lack of plasmalogens, although also other ether phospholipids, like alkylphospholipids (lacking the vinyl ether bond characteristic for plasmalogens) or platelet-activating factor, are depleted in all types of RCDP and, in case of RCDP type 1, elevated plasma levels of phytanic acid have been detected [186]. Irrespective of the affected gene, all RCDP patients share common symptoms. The most typical are the eponymous shortening of the proximal long bones (rhizomelia) and epiphyseal stippling (chondrodysplasia punctata) as well as congenital cataracts, joint contractures and growth and developmental retardation [187]. The severity of the disease varies remarkably, depending strongly on the residual activity of the affected enzyme and, thus, the level of plasmalogens [188–191]. The most severe form of the disease leads to lethality within the first years of life, often due to respiratory failure. In contrast, patients with a less severe disease course present with only some of the characteristic symptoms and can survive into young adulthood [192–194]. In human ether phospholipid deficiency, multiple pathological features affect brain development and function. Mental disability and delayed motor development are hallmarks of RCDP. However, the extent to which the brain is affected varies remarkably between patients and many of the symptoms are restricted to the severer forms of the disease. Frequent delay in brain development is further reflected by the finding of microcephaly in many RCDP cases. Also epileptic seizures are very common but non-specific; seizure type and frequency vary considerably and even affected children with multiple seizure types have been reported [191,192]. The age of onset of seizure activity is reportedly higher in milder cases of RCDP [191].

MRI examination in children with RCDP has revealed varying pathologic features, although some cases, usually with a milder phenotype, with unremarkable MRI results are also found [195]. Consistently, most reports describe enlargement of ventricles and the subarachnoid space, abnormalities in white matter signal intensity and delayed supratentorial white matter myelination with frequent involvement of the parieto-occipital area [195–200]. In line with myelination defects also in the PNS, peripheral neuropathy has been reported in a clinical subset of RCDP patients [201]. The severe form of the disease is usually accompanied by progressive cerebellar atrophy [195], which is caused by a pronounced loss of Purkinje cells and, to a lesser extent, other cell types in the cerebellum [202]. Thus, in addition to peroxisomal β-oxidation, also ether lipid biosynthesis appears to be crucial for cerebellar development and function. Originally, it was assumed that increased levels of phytanic acid contribute to the pathogenesis in the cerebellum [52,202]. However, this hypothesis is strongly weakened by the presence of cerebellar atrophy in a case of RCDP type 3, in which ether lipid deficiency is the only metabolic defect, and, conversely, by the absence of cerebellar atrophy in a case with particularly high phytanic acid levels [191]. Sporadically, also other brain malformations have been observed, like temporal atrophy [197], agenesis of the corpus callosum [203], polymicrogyria [200], and pachygyria [204,205]. Neuronal migration defects never reach the extent of those observed in Zellweger syndrome (see Fig. 2A), but several cases with dysplastic olivary bodies have been reported [202,206]. These findings emphasize the fact that multiple peroxisomal functions are required for proper development of the brain. In addition to the brain pathology, patients suffering from the severe form of RCDP often develop stenosis of the spinal canal (cervical stenosis) [195,207]. From a metabolic point of view, MR spectroscopy of the brain has shown increased levels of *myo*-inositol, a marker for gliosis, in line with previous reports of gliosis in autopsy cases of RCDP [52,208]. MR spectroscopy has also revealed elevated levels of mobile lipids, most likely caused by accumulation of long-chain acyl-CoAs, as well as a reduction of choline and the presence of acetate [199,209].

More insight into the pathomechanisms of ether lipid deficiency has been gained from the generation of ether lipid-deficient mouse models. Currently, gene KO mouse models exist for RCDP types 1–3 (*Pex 7, Gnpat* and *Agps* KO mice, respectively) [210–212], of which the first two models have been extensively characterized. Recently, also a mouse model with inducible inactivation of alkyl/acyl-dihydroxyacetone phosphate reductase (AADHAPR; also named peroxisomal reductase activating PPARγ, PexRAP), the enzyme catalyzing the third step in ether lipid biosynthesis (following the DHAPAT and ADHAPS reactions) and shown to be located at the outer face of the peroxisomal membrane [213], was generated [214]. Furthermore, hypomorphic mouse models with residual transcript levels of the *Pex7* [215] or the *Agps* [216] gene and, consequently, residual levels of ether lipids have been described, which mimic the milder form of the human disease. Many of the phenotypic brain abnormalities described in these ether lipid-deficient mice resemble the observations in humans, with the advantage of animal models being the opportunity to elucidate the underlying molecular processes in more detail. Several studies have reported hypomyelination in different brain areas (neocortex, corpus callosum, cerebellum) of ether lipid-deficient mice [217,218], but no progressive demyelination as seen in several mouse models of Zellweger syndrome [73]. Also myelin of the PNS is affected in ether lipid-deficient mice. Deficiencies in myelination as well as Schwann cell development and differentiation are found [219] resulting in peripheral neuropathy, as judged by reduced motor neuron conduction velocity [220]. Remarkably, hypo- and dysmyelination in the CNS of ether lipid-deficient animals is accompanied by slight loss of axons and mild astrogliosis in some brain areas, whereas microgliosis, which has been reported in several RCDP cases, and the induction of inflammatory cytokines appear to be less pronounced [73]. However, neuroinflammation with marked microglia activation was observed upon combined deficiency of *Pex7* and *Abcd1* in 11-months-old mice of mixed background (Swiss Webster and C57BL/6J:129S1) [220] suggesting that ether lipid deficiency has little effect on immune cell activation under basal conditions but more drastic consequences in the presence of an additional immunostimulatory factor. This concept is also supported by the finding that plasmalogens suppress microglia activation after systemic injection of lipopolysaccharides (LPS) in mice [221,222]. Furthermore, in murine cell lines, plasmalogens seem to counteract neuronal death elicited by serum starvation [223,224] by utilizing a process, which has been reported to involve protein kinase B (AKT) signaling [224].

Cerebellar pathology is a striking feature in human cases of ether lipid deficiency and has received special attention in the study of the corresponding mouse models. Concordantly, foliation defects with underdevelopment of fissures, particularly affecting foliae VI and VII, have been reported in *Gnpat* KO mice at different postnatal stages [218,225]. Also, a migration defect of granule cell precursors [218] and increased numbers of apoptotic cells in the external granule layer [225] were found. Furthermore, hypomyelination in cerebellum (and also in cortical areas) of *Gnpat* KO mice was accompanied by changes in the architecture of the nodes of Ranvier resulting in a delay in the propagation of action potentials [218]. Microscopy studies revealed structural abnormalities in the innervation of Purkinje cells by parallel fibers and climbing fibers as well as axonal swellings with accumulation of smooth ER-like structures in Purkinje cells [218].A detailed review of cerebellar pathology in the context of ether lipid deficiency and other peroxisomal disorders has been published recently [226].

In the first description of the *Pex7* KO mouse, neuronal migration defects in the neocortex of mutant embryos were reported; however, these were much less pronounced than the migration deficits in mice completely lacking peroxisomes (e.g. *Pex5* KO mice) [210]. Remarkably, although similar techniques were used, no such alterations could be detected in the brains of *Gnpat* KO mice [225], pointing towards a role of phytanic acid in the development of these migration defects or potential differences in the background strains of the mice used in the different studies. Da Silva and coworkers also speculated that a not yet identified peroxisomal protein harboring a PTS2 (and therefore being dependent on PEX7) may be responsible for these apparently conflicting results [227]. By making use of the progress in the elucidation of PTS2 structure requirements [228], future studies may substantiate this idea.

Involvement of the visual system is another typical feature of RCDP that has been extensively studied in mouse models. Lens anomalies, particularly bilateral cataracts, have been reported in all ether lipid-deficient mouse models, including the hypomorphic mice. Furthermore, hypoplasia of the optic nerve, microphtalmia, a persistent hyaloid artery [211] and abnormal vascularization [229] were detected in *Gnpat* KO mice. These ocular abnomalities have been covered in detail in previous reviews [217,230].

So far, the molecular bases of mental disability in RCDP and the nervous system pathology upon ether lipid deficiency in mice and men have only been partially unraveled. Recently, a defect in the activation and downstream signaling of AKT was proposed to be responsible for the myelination deficit in the PNS of ether lipid-deficient animals [219]. It remains to be determined, whether a similar disturbance affects the CNS as well. Plasmalogen-deficient myelin might be more prone to oxidative damage [231]; however, this idea is weakened by the observations of reduced rather than elevated levels of malondialdehyde and no substantial change in other oxidative stress markers in the brains of ether lipid-deficient mice [40,73]. Studies in synaptosomes isolated from cortex, mimicking the process of synaptic transmission, have revealed a decrease in calcium-dependent release of the neurotransmitters glutamate and acetylcholine in *Gnpat* KO mice [40]. This goes along with decreased ATP content and an inability of synaptosomal respiration to adapt to the higher energy requirements of depolarization, thereby offering a potential explanation for the defects in synaptosomal neurotransmitter release [40] (Fig. 3B). Alternatively, changes in membrane properties caused by plasmalogen deficiency might play a role (Fig. 3B). The lack of ethanolamine plasmalogens (by far the most abundant plasmalogen in the brain) is strictly compensated by the structurally similar phospholipid phosphatidylethanolamine [40,232]. However, the characteristic biophysical properties conferred by plasmalogens [233–235] could be essential for neurotransmission, which involves repeated membrane fusion and constriction processes (Fig. 3B). Furthermore, it has been suggested that deficiency in plasmalogens impairs membrane rafts (formerly termed lipid rafts) [211], small membrane domains that organize various cellular processes [236,237] and are enriched in plasmalogens [238]. However, the impact of ether lipid deficiency on neurotransmission *in vivo* and the underlying molecular mechanism still have to be elucidated in greater detail.

No characterization of the nervous system in hypomorphic mouse models of RCDP has been published so far, which could be due to the milder phenotype of these mice, leaving the nervous system largely unaffected.

## 6 The contribution of peroxisomes to more common neurological disorders

Besides the fact that inherited peroxisomal disorders have drastic consequences for the nervous system, a dysfunction of peroxisomes or a dysregulation of peroxisomal metabolites has also been described in a variety of other, more common, neurological diseases.

### 6.1 Involvement of D-amino acid oxidase in amyotrophic lateral sclerosis and schizophrenia

Across evolution, D-amino acid oxidase (D-AAO, DAO) serves as a tool to access the nutritional supply; however, in the brain, this enzyme and its more specific counterpart D-aspartate oxidase (DDO) exert a regulatory function in modulating the amount of the neuroactive D-amino acids D-serine and D-aspartate, respectively [239]. DAO is peroxisomal [13,240], contains a functional PTS1 motif [241] and interacts with PEX5 [242] and also human DDO was shown to be located in peroxisomes [243,244]. These enzymes are flavin adenine dinucleotide (FAD)-containing flavoenzymes that oxidize certain amino acids, thereby generating H_2_O_2_ as side product. DAO activity is abundant in various human brain areas, but in the murine brain, some of the corresponding regions contain markedly lower activity [245].

D-Serine binds to a specific extracellular site on the N-methyl-D-aspartate (NMDA) receptor, which responds to glutamate as neurotransmitter, and further increases the signaling strength [246]. In the brain, D-serine is generated in astrocytes and released into the synaptic cleft [247], but also taken up from the synaptic cleft and degraded in astrocytic peroxisomes (see Fig. 3B).

Genetic linkage between the *DAO* locus and schizophrenia was first described by Chumakov and collaborators [248] and has since been observed by several groups. In a later study, also elevated activity of DAO was linked to schizophrenia [249], corresponding to reduced D-serine levels and NMDA receptor hypoactivity. An increased activity of DAO has been found in the cortex of patients [250] and the protein level was increased in cerebellum and cortex [251], while reduced levels of D-serine were observed in the cerebrospinal fluid of patients [252]. Thus, inhibitors of DAO have been suggested as a therapeutic option for schizophrenia [253]. However, it is unclear, whether the relevant DAO activity is entirely peroxisomal in the astrocytes of patients.

Furthermore, a mutation in DAO was recently linked to a familiar form of amyotrophic lateral sclerosis (ALS) [254], which is a fatal human disease with neurodegenerative aspects affecting predominantly motor neurons. The link to D-amino acid metabolism was supported by the finding that in a mouse model for the familial form of ALS (SOD1^G93A^), DAO activity in the spinal cord was reduced and, consequently, D-serine levels were increased [255].

### 6.2 The link between ether lipid biosynthesis and autism

In 2013, a study applying whole exome sequencing identified a link between mutations in *PEX7* (RCDP type 1) and autism spectrum disorder, a range of neurodevelopmental conditions characterized by deficits in communication and social interaction and repetitive behavior, in a family with three affected children [256]. This observation supports previous work showing an association between single nucleotide polymorphisms in the *PEX7* gene and autism [257]. Prompted by their findings, the authors reviewed previously reported RCDP cases for potential signs of autism and found two further patients, which had later been diagnosed with neurodevelopmental conditions (one with autism, the other with attention deficit hyperactivity disorder) [256]. Additional consolidation of the proposed link between plasmalogen deficiency and autism comes from the finding of reduced levels of plasmalogens in plasma and red blood cells of autistic patients [258,259]. The mechanism, by which these two phenomena are connected, is still fully unexplored. Autistic features have, so far, not been regarded as a typical symptom of RCDP (or other peroxisomal disorders), although one similar case was already mentioned in 1999 [260]. The reasons for this might be that many children affected by the disease do not reach a stage, in which symptoms of autism manifest, and that treatment of patients with RCDP focuses on other, more vital aspects. However, in the future, clinicians may pay more attention to signs of autism in RCDP patients, especially those suffering from the milder form of the disease, which should help to substantiate a relationship between deficiency in plasmalogens and autism.

### 6.3 Peroxisomes and Alzheimer’s disease

Several links have been found between peroxisomes and Alzheimer’s disease (AD), the most common form of dementia affecting several millions of people worldwide. Most prominently, a role of plasmalogen deficiency in the etiology of AD is considered. Many studies have confirmed a severe depletion of ethanolamine plasmalogens (ethanolamine is by far the most abundant plasmalogen head group in the CNS), in *post mortem* brain tissue of AD patients [261–266]; but also contradictory results exist [267]. The decrease in brain plasmalogens emerges early in the disease course [262,268] and, at least in gray matter, corresponds well with the deterioration of cognitive function [262]. Although, to a lesser extent, also associated with normal aging [269], depletion of brain plasmalogens appears to be rather specific for AD and not a general feature of neurodegeneration. Similar abnormalities could not be found in several other neurodegenerative diseases like Parkinson’s disease or Huntington’s disease [270]. Remarkably, deficiency of ethanolamine plasmalogens was also repeatedly detected in peripheral blood of AD patients [271] rendering these lipids potential as biomarkers for early and easy detection of the disease [272].

The origin of plasmalogen deficiency in AD is currently still unknown. Grimm and coworkers suggested that a dysregulation of *AGPS* expression by the amyloid precursor protein intracellular domain and oxidative damage by amyloid-beta (Aβ) peptides lead to instability and loss of activity at the protein (ADHAPS) level causing decreased plasmalogen biosynthesis in AD [266]. Others speculate that an Aβ-mediated increase in the activity of plasmalogen-selective phospholipase A2 (PLA2), as detected in certain brain regions of AD patients [273], depletes plasmalogens, thereby leading to excessive vesicular fusion and, finally, synaptic failure [274]. Other alternative explanations include increased oxidation of plasmalogens, in line with their proposed role as radical scavengers or excessive membrane degradation. However, also impaired generalized function of peroxisomes could contribute to the disturbance of plasmalogen homeostasis. This hypothesis is supported by findings of altered levels also of other peroxisomal metabolites in the context of AD. For example, increased VLCFA levels were detected in cortical tissues [263,264] and in peripheral blood [275] of AD patients (although the latter results still require confirmation). Also, a decrease in DHA, whose endogenous *de novo* production requires peroxisomes, in brain and liver [276] and a regional decrease in catalase activity in the temporal lobe [277] were reported. Furthermore, our group has previously identified an accumulation of peroxisomes in the somata of neurons in the gyrus frontalis of AD patients accompanied by a lack of peroxisomes in dendrites with abnormally phosphorylated Tau protein, which might prevent the transport of peroxisomes into these processes [263]. These results are complemented by studies in mouse models of AD, which imply that the number and protein content of peroxisomes are strongly modulated by the disease course [278,279], and that these alterations are possibly triggered by excessive oxidative stress and/or mitochondrial dysfunction.

As the etiology of AD is still unresolved, also the role of peroxisomal dysfunction in the disease process is debated. It might be speculated, though, that a decrease in peroxisomal activity, even if not the primary cause of the observed pathology, aggravates oxidative stress and neurodegeneration in the AD brain. This fits the observation that treatment with a PPARα agonist, presumably by increasing the number of peroxisomes, protects cultured rat hippocampal neurons from Aβ-mediated cell death [280]. In line with this, based on the changes in expression of peroxisomal enzymes in rat cortical neuron cultures upon different treatment regimens with Aβ, it has also been hypothesized that peroxisomes represent an important defense mechanism against oxidative stress triggered by Aβ [281]. In addition, elevated levels of VLCFA and reduced levels of plasmalogens, both markers of peroxisomal dysfunction, have been suggested to stimulate the production of Aβ peptides [282,283]. Detailed reviews covering the potential roles of plasmalogen depletion [284] or peroxisomal impairment [285] in the context of AD have been published in recent years.

An independent connection between peroxisomes and AD is provided by insulin-degrading enzyme (IDE; see Fig. 1), a Zn^2+^-dependent endopeptidase, whose name-giving enzymatic activity was described already in 1949 [286]. This peptidase can degrade a variety of physiologically relevant peptides reaching from glucagon via Aβ to insulin-like growth factors (for review see [287]). IDE has been found in peroxisomes of cultured cells upon overexpression; and in rat liver, a fraction of the protein was localized to peroxisomes [288–291]. Moreover, this peptidase can degrade N-terminal peptides derived from PTS2-carrying pre-proteins upon processing inside peroxisomes [288]. However, IDE was also described at many other subcellular locations such as mitochondria [292], the nucleus [293], the plasma membrane [294] or the extracellular space [295].

Because IDE can also degrade Aβ [295], this protease has been suggested as a candidate for a modulatory function in the pathophysiology of AD [296]. In line with an important contribution of Aβ degradation by IDE to the pathology of AD, an early genetic investigation described a genetic link between late-onset AD and a region of chromosome 10 that includes the *IDE* locus [297]. However, some subsequent studies using various single nucleotide polymorphisms found evidence for a linkage while others did not. Moreover, a recent meta-analysis could not confirm indications for a contribution of individual single nucleotide polymorphisms [298]; and in genome-wide association studies, the locus has not been identified [299]. In the mouse, the deletion of *Ide* (by gene KO) was accompanied by reduced degradation of insulin and higher levels of Aβ in the brain [300]. Moreover, the neuron-specific ectopic expression of *IDE* in a mouse model for AD (APPSwe/Ind) [301] was sufficient to relieve the burden of amyloid plaque-related pathology [302]. Consequently, the upregulation of IDE activity has been amply suggested for therapeutic purposes. However, it has to be stressed that the occurrence of IDE in various subcellular compartments renders the attribution of specific contributions of IDE functions to one location like peroxisomes rather difficult.

### 6.4 Peroxisomes and other neurological diseases

In recent years, with increasing progress in lipidomic techniques, changes in the amounts of plasmalogens either in the brain or in the periphery have been described in a variety of neurological diseases. These include Parkinson’s disease [303], schizophrenia [304,305], Down syndrome [306,307], Pelizaeus–Merzbacher disease [308], and lysosomal storage disorders like Gaucher’s disease [309] or neuronal ceroid lipofuscinosis [310]. However, in many cases the detected changes might be too small to be physiologically relevant and are likely to be counteracted by compensatory lipid changes [232]. Additionally, changes of plasmalogen levels may be secondary to the plethora of degenerative and pathologic processes in these diseases. Special caution is warranted for the interpretation of altered levels of plasmalogens in peripheral blood, as even in the milder forms of peroxisomal disorders some cases are known with normal plasmalogen levels in plasma (see Section 5.1.1.). Therefore, only limited conclusions about pathology can be drawn from these values; this can also be deduced from the observation that lipid levels in blood and brain in disease often correlate poorly [311].

It should be noted in the context of peroxisomes and neurodegenerative disorders that in all three Zellweger mouse models (*Pex2, Pex5* and *Pex13* deficiency [60–62]), increased α-synuclein oligomerization was observed in brain tissues [312]. This is of particular interest, as in Parkinson’s disease and related synucleinopathies, the normally presynaptic protein α-synuclein aggregates intraneuronally to form Lewy bodies, the neuropathological hallmark of these diseases. When α-synuclein was overexpressed in murine fibroblasts, the oligomerization and phosphorylation of α-synuclein was markedly higher in *Pex5*-deficient cells than in control fibroblasts [312]. In this study, it was suggested that α-synuclein oligomerization and aggregation correlate with lipid alterations rather than with mitochondrial dysfunction or oxidative stress.

Based on the observations that in murine experimental autoimmune encephalomyelitis (EAE) several peroxisomal functions appear to be impaired [313] and that peroxisome deficiency goes along with severe neuroinflammation, a contribution of peroxisomal dysfunction to the pathology in multiple sclerosis, a chronic inflammatory demyelinating disease of the CNS, was postulated [314]. Gray and coworkers further supported their hypothesis by showing decreased expression of *ABCD3* mRNA together with a reduction of ABCD3/PMP70 immunoreactivity in gray matter within and outside of lesions, as well as a slight elevation of VLCFA levels in *post mortem* cortical gray matter from MS patients [314]. Contrary to the idea of a general impairment of peroxisomal functions, the results of a recent report indicate a tendency towards increased plasmalogen levels in the serum of multiple sclerosis patients [315]. However, as discussed above, peripheral lipid levels may be of limited relevance for the interpretation of pathological processes in the brain.

## 7 Concluding remarks

The functionality of the nervous system critically depends on the ability of peroxisomes to provide biosynthetic intermediates and to degrade undesired compounds that interfere with brain formation, brain function or brain preservation. Moreover, peroxisomes participate in the maintenance of metabolites in the appropriate concentration ranges (e.g., D-amino acid or ROS levels). A lack of each of these functions causes structural abnormalities of the brain, in particular in the cortex or the cerebellum, defects in the intercellular communication of neurons affecting electrical propagation rates along the axon and synaptic transmission efficiency or inappropriate onset of aging and inflammatory processes.

The spectrum of neurological symptoms observed in patients suffering from PBD or single enzyme and transporter deficiencies is surprisingly broad. This variability can be traced back to: (i) residual activity of the affected protein causing milder pathologies (PBD), (ii) the genetic background of patients rendering them more susceptible or resilient, or (iii) environmental factors ranging from disease-inducing occurrences to protection by nutrition deprived of detrimental compounds (Refsum disease). This implies that patients with mild variants of PBD might even escape correct diagnosis. Moreover, the observations that peroxisomes tightly interact with other organelles and that peroxisomal dysfunction secondarily affects their functionality suggest that patients suffering from inherited diseases originating from peroxisomes may benefit from therapeutic approaches targeting such secondary sites.

Finally, increasing evidence indicates that peroxisomes also exert a modulatory role in more common neurodegenerative disease such as AD, autism, ALS or schizophrenia. This is partially supported by linkage analyses, but also by comparative measurements of enzymatic activities, expression levels of mRNAs or proteins or changes in metabolite concentrations linked to peroxisomal functions between cohorts of patients and healthy controls. Future work applying steadily improving bioanalytical tools will help to decipher the relationship between the accumulation or deprivation of biomolecules linked to peroxisomes (e.g., DHA, plasmalogens or D-amino acids) and certain physiological or pathophysiological conditions.

## Supplementary Material

The Transparency document associated with this article can be found, in online version,

Transparency document

## Figures and Tables

**Table 1:** Genetic basis of peroxisomal disorders.

Gene	Protein	Disease	Phenotype MIM	Reference
***Peroxisome biogenesis disorders***		**Zellweger syndrome spectrum disorder**		
*PEX1*	Peroxin 1 (PEX1)	Zellweger syndrome,	214100	[316]
		neonatal adrenoleukodystrophy, infantile Refsum disease	601539
*PEX2*	Peroxin 2 (PEX2)	Zellweger syndrome,	614866	[46]
		infantile Refsum disease	614867	[317]
*PEX3*	Peroxin 3 (PEX3)	Zellweger syndrome	614882	[318]
*PEX5*	Peroxin 5 (PEX5)	Zellweger syndrome,	214110	[319]
		neonatal adrenoleukodystrophy	202370
*PEX6*	Peroxin 6 (PEX6)	Zellweger syndrome,	614862	[320]
		neonatal adrenoleukodystrophy, infantile Refsum disease	614863	[321]
*PEX10*	Peroxin 10 (PEX10)	Zellweger syndrome,	614870	[322]
		neonatal adrenoleukodystrophy	614871
*PEX12*	Peroxin 12 (PEX12)	Zellweger syndrome,	614859	[323]
		neonatal adrenoleukodystrophy, infantile Refsum disease	266510	[324]
*PEX13*	Peroxin 13 (PEX13)	Zellweger syndrome,	614883	[325]
		neonatal adrenoleukodystrophy	614885	[326]
*PEX14*	Peroxin 14 (PEX14)	Zellweger syndrome	614887	[327]
*PEX16*	Peroxin 16 (PEX16)	Zellweger syndrome	614876	[328]
		Mild Zellweger syndrome spectrum disorder	614877	[58]
*PEX19*	Peroxin 19 (PEX19)	Zellweger syndrome	614886	[329]
*PEX26*	Peroxin 26 (PEX26)	Zellweger syndrome,	614872	[330]
		neonatal adrenoleukodystrophy, infantile Refsum disease	614873
*PEX11β*	Peroxin 11β (PEX11β)	Mild Zellweger syndrome spectrum disorder	614920	[331,332]
*PEX7*	Peroxin 7 (PEX7)	**Rhizomelic chondrodysplasia punctata type 1**	215100	[176–178]
			614879	[190]
***Single peroxisomal enzyme and transporter deficiencies***				
**Fatty acid β-oxidation**				
*ACOX1*	Acyl-CoA oxidase 1 (ACOX1)	Acyl-CoA oxidase deficiency	264470	[333]
*HSD17B4*	D-Bifunctional protein^a^	D-Bifunctional protein deficiency	261515	[334]
		Perrault syndrome 1	233400	[85]
*SCP2*	Sterol carrier protein 2 (SCP2)^b^	Sterol-carrier-protein X deficiency	613724	[102]
*AMACR*	α-Methylacyl-CoA racemase	α-Methylacyl-CoA racemase deficiency	614307	[93]
		Congenital bile acid synthesis defect 4	214950
*ABCD1*	ATP-binding cassette transporter, subfamily D, member 1 (ABCD1)	X-linked adrenoleukodystrophy	300100	[108]
*ABCD3*	ATP-binding cassette transporter, subfamily D, member 3 (ABCD3)	ATP-binding cassette transporter, subfamily D, member 3 deficiency	616278	[335]
**Fatty acid** α**-oxidation**				
*PHYH/PAHX*	Phytanoyl-CoA hydroxylase (PHYH, PAHX)	Refsum disease	266500	[170,336]
**Ether phospholipid biosynthesis**				
*GNPAT*	Dihydroxyacetone phosphate acyltransferase (DHAPAT)	Rhizomelic chondrodysplasia punctata type 2	222765	[179]
*AGPS*	Alkyl-dihydroxyacetone phosphate synthase (ADHAPS)	Rhizomelic chondrodysplasia punctata type 3	600121	[180]
*FAR1*	Fatty acyl-CoA reductase 1 (FAR1)	Rhizomelic chondrodysplasia punctata type 4/peroxisomal fatty acyl-CoA reductase 1 deficiency	616154	[183]
*PEX5*	Peroxin 5 long isoform (PEX5L)	Rhizomelic chondrodysplasia punctata type 5	-	[185]
**Bile acid maturation**				
*BAAT*	Bile acid CoA:amino acid N-acyl-transferase (BAAT)	Familiar hypercholanemia/bile acid-CoA: amino acid N-acyltransferase deficiency	607748	[337]
**Glyoxylate metabolism**				
*AGXT*	Alanine-glyoxylate aminotransferase (AGXT, AGT)	Primary hyperoxaluria type I	259900	[338]
**Hydrogen peroxide metabolism**				
*CAT*	Catalase	Acatalasemia	614097	[339]
**Others**				
*ALDH3A2*	Fatty aldehyde dehydrogenase (FALDH)^c^	Sjögren–Larsson syndrome	270200	[340]
*DAO*	D-Amino acid oxidase (DAO, DAAO)	Amyotrophic lateral sclerosis	105400	[254]

a Alternative names: 17-β-hydroxysteroid dehydrogenase IV (HSD17B4)/multifunctional protein 2 (MFP2).

b Alternative name: sterol carrier protein X (SCPX).

c Two isoforms are known residing in peroxisomes and the ER, which precludes attribution of the disease to a particular variant.

## Footnotes

1 Abbreviations: Aβ, amyloid-β; ABC, ATP-binding cassette; ACAA, acetyl-CoA acyltransferase; ACOX, acyl-CoA oxidase; AD, Alzheimer’s disease; ADHAPS, alkyl-dihydroxyacetone phosphate synthase; ALS, amyotrophic lateral sclerosis; AMACR, 2-methylacyl-CoA racemase; AMN, adrenomyeloneuropathy; CALD, cerebral X-ALD; CNS, central nervous system; CT, computed tomography; DAO, D-amino acid oxidase; DBP, D-bifunctional protein; DDO, D-aspartate oxidase; DHA, docosahexaenoic acid; DHAPAT, dihydroxyacetone phosphate acyltransferase; DHCA/THCA, di-/trihydroxycholestanoic acid; ER, endoplasmic reticulum; FAR, fatty acyl-CoA reductase; IDE, insulin-degrading enzyme; KO, knockout; MRI, magnetic resonance imaging; PBD, peroxisome biogenesis disorders; PEX, peroxin; PHYH, phytanoyl-CoA hydroxylase; PMP, peroxisomal membrane protein; PNS, peripheral nervous system; PTS, peroxisomal targeting signal, RCDP, rhizomelic chondrodysplasia punctata; ROS, reactive oxygen species; SCPx, sterol carrier protein X; VLCFA, very long-chain fatty acids; X-ALD, X-linked adrenoleukodystrophy.

2 When MIM numbers are indicated within this manuscript, we always refer to phenotype MIM numbers (online source: www.omim.org) characterizing the respective disorder.
